# Response of pollinator taxa to fire is consistent with historic fire regimes in the Sierra Nevada and mediated through floral richness^†^

**DOI:** 10.1002/ece3.10761

**Published:** 2023-12-14

**Authors:** Gina L. Tarbill, Angela M. White, Rahel Sollmann

**Affiliations:** ^1^ Pacific Southwest Research Station USDA, Forest Service Davis California USA; ^2^ Wildlife, Fish, & Conservation Biology University of California, Davis Davis California USA; ^3^ Department of Ecological Dynamics Leibniz Institute for Zoo and Wildlife Research Berlin Germany

**Keywords:** bees, burn severity, butterfly, fire, hummingbird, pollinator

## Abstract

Many fire‐prone forests are experiencing wildfires that burn outside the historical range of variation in extent and severity. These fires impact pollinators and the ecosystem services they provide, but how the effects of fire are mediated by burn severity in different habitats is not well understood. We used generalized linear mixed models in a Bayesian framework to model the abundance of pollinators as a function of burn severity, habitat, and floral resources in post‐fire, mid‐elevation, conifer forest, and meadow in the Sierra Nevada, California. Although most species‐level effects were not significant, we found highly consistent negative impacts of burn severity in meadows where pollinators were most abundant, with only hummingbirds and some butterfly families responding positively to burn severity in meadows. Moderate‐severity fire tended to increase the abundance of most pollinator taxa in upland forest habitat, indicating that even in large fires that burn primarily at high‐ and moderate‐severity patches may be associated with improved habitat conditions for pollinator species in upland forest. Nearly all pollinator taxa responded positively to floral richness but not necessarily to floral abundance. Given that much of the Sierra Nevada is predicted to burn at high severity, limiting high‐severity effects in meadow and upland habitats may help conserve pollinator communities whereas low‐ to moderate‐severity fire may be needed in both systems.

## Graphical abstract

Many dry forests in the western United States are experiencing wildfires that burn outside the natural range of variation in extent and severity. We investigated the impacts of burn severity, habitat, and floral resources on pollinator abundance in conifer forest (upland) and meadow habitats in a megafire in the Sierra Nevada, California. Most pollinator taxa did not repond significantly to burn severity, however there were trends of peak abundance at moderate severity fire in upland habitat and decreasing abundance for most taxa with increasing burn severity in meadow habitat. Floral richness, but not abundance, tended to increase pollinator abundance significantly. Prioritizing meadows for fuel reduction and restoration and supporting floral diversity may help conserve pollinator communities threatened by high‐severity fire.

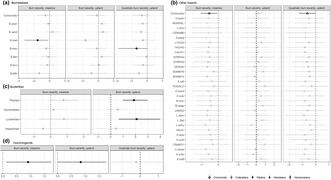

## 1 INTRODUCTION

Many historically pyrodiverse regions are becoming homogenized by the combined effects of climate change, fire suppression, and land use changes (Bowman et al., 2011; Hagmann et al., 2021; Pausas & Fernández‐Muñoz, 2012; Seidl et al., 2016). Perturbations to historic fire regimes may increase the severity, frequency, or extent of contemporary fires, with cascading effects on biodiversity and ecosystem function (Williams, 2013; Stephens et al., 2014; Seidl et al., 2016). For example, fire severity has significantly increased in many fire‐prone regions of the world that historically burned at low or moderate severity (Flannigan et al., 2013; Jones et al., 2022; Stephens et al., 2014). The impacts of high‐severity fire on these systems include lower recruitment and establishment of native plant species (Collins et al., 2017; Etchells et al., 2020), shifts in vegetation communities (Parks et al., 2019; Scheffer et al., 2001), and loss of plant diversity (Miller et al., 2018; Weeks et al., 2023) and ecosystem services (Adams, 2013; Benavides‐Solorio & MacDonald, 2001; Hurteau & Brooks, 2011). These changes may further alter disturbance regimes through positive feedback (Pausas & Keeley, 2019). Research on the effect of changed fire regimes, however, has focused on plant communities, and similar information is sparse or lacking for most animals, even those highly dependent on plants, such as pollinators (Jager et al., 2021; White & Long, 2019).

Pollinator community structure is strongly associated with the abundance, diversity, and resource quality of flowering plants, suggesting that fire regime shifts that impact forest structure and nutrient recycling are likely to impact pollinators, with impacts varying across taxa (Fowler et al., 2016; Glenny et al., 2023; Potts, Vulliamy, Dafni, Ne'eman, O'toole, et al., 2003). Fires may affect pollinators directly by consuming nesting or diapausing organisms or indirectly by changing foraging or nesting substrates (Cane & Neff, 2011; Koltz et al., 2018; New, 2014). Recent reviews on the effects of fire on pollinators found positive responses in abundance, but there were differences among taxonomic groups: although bees (Hymenoptera: Anthophila) tended to increase in abundance after fire, the responses were mixed for flies (Diptera), butterflies (Lepidoptera), beetles (Coleoptera), and hummingbirds (Trochilidae; Alexander et al., 2020; Carbone et al., 2019; Mason Jr et al., 2021). Differences among taxonomic groups may be due to life history traits or dependence on particular resources or habitat features (Bouget et al., 2013; Fleishman, 2000; Häussler et al., 2017). Species like bees, which depend on pollen and nectar for nutrition in all active life stages, may be most influenced by the availability of floral resources (Vaudo et al., 2015). Other taxa with life stages that depend on different resources, such as butterflies with specific larval host plants (Dennis & Shreeve, 1988; Fleishman, 2000), or flower‐feeding beetles (e.g., Cerambycidae) that develop in dead or decaying wood (Bouget et al., 2013; Grove, 2002), may be more closely associated with mesic habitats or closed‐canopy habitats (O'Neill et al., 2008), respectively. Long‐lived or migratory species, such as hummingbirds, may also be influenced by site fidelity or resource reliability (Moore & Aborn, 2000; Russell et al., 1994). Across taxonomic groups, mixed responses to fire may also be due to non‐linear relationships with burn severity (Lazarina et al., 2019; Mason Jr et al., 2021), complex interactions among burn severity and fire history (Ponisio et al., 2016), or deviations from the historic fire regime (Koltz et al., 2018).

As the frequency and intensity of fire vary with vegetative structure, pollinator responses to fire will likely be mediated by habitat type. For example, in closed‐canopy fire‐prone habitats, the abundance and diversity of pollinators tend to be highest after moderate‐severity fire (Lazarina et al., 2019; Ponisio et al., 2016). Under these structural conditions, moderate‐severity fire creates canopy gaps that support more diverse and abundant floral resources (Richter et al., 2019) and nesting substrates (Brokaw et al., 2023; Felderhoff et al., 2023; Potts, Vulliamy, Roberts, et al., 2005), and increase habitat heterogeneity (Martin & Sapsis, 1992, Parr & Andersen, 2006), thereby reducing the distances pollinators need to travel to acquire resources (Jha & Kremen, 2013). However, when burned at high‐severity, fire can limit source populations and dispersal of pollinators (Cane & Neff, 2011; Galbraith et al., 2019a, 2019b; Lazarina et al., 2019) or vegetative recovery (DeBenedetti & Parsons, 1984; Potts, Vulliamy, Dafni, Ne'eman, & Willmer, 2003), particularly as high‐severity patch size increases, while unburned closed‐canopy forests lack floral resources (Burkle et al., 2015; Potts, Vulliamy, Dafni, Ne'eman, O'toole, et al., 2003; Rodríguez & Kouki, 2017). In contrast, open habitats are dominated by herbaceous cover, and fires consume nearly all biomass, but vegetative recovery tends to happen quickly (DeBenedetti & Parsons, 1979; Pereira et al., 2016; Zouhar, 2021). Fire in these systems is more likely to alter community composition and flowering phenology than diversity or abundance of plants (Mola & Williams, 2018; Tarbill, 2022), but high‐severity fire may have negative impacts on seedbanks, soil, and pollinator recolonization.

In this study, we examine the response in pollinator abundance to a large‐scale, high‐severity fire in the Sierra Nevada Mountains of California, USA, where human perturbations to the fire regime have led to an increase in frequency and size of high‐severity fire. Specifically, we investigate the effect of burn severity and availability of floral resources in closed‐canopy (henceforth, upland) and open (henceforth, meadow) habitats. We expected that fire would increase pollinator abundance in upland habitat by increasing the availability of foraging and nesting resources, with moderate‐severity upland habitat having a higher pollinator abundance than high‐severity upland. In contrast, we hypothesized that the abundance of pollinators in meadows is likely to decrease with increasing burn severity. Because pollinators are highly dependent on floral resources, we expected pollinator abundance to be positively associated with increasing floral abundance and richness, regardless of habitat. Specifically, we expected bees to respond most strongly to floral resources, as they are highly dependent on pollen and nectar, with weaker responses for wasps due to their predatory nature. We hypothesized that butterflies would be more abundant in meadows than uplands because many larval host plants are dependent on mesic habitat (Dennis & Shreeve, 1988; Fleishman, 2000). Similarly, we expected hummingbird abundance to be greater in meadows than upland habitat due to the reliability of meadows as a source for floral resources (Moore & Aborn, 2000; Russell et al., 1994). Finally, because flower‐visiting beetles and flies depend on dead and decaying wood in larval stages, we expected them to be more abundant in upland relative to meadow habitat and positively associated with burn severity. True bugs (Hemiptera) are typically considered herbivores rather than pollinators (but see Garcia et al., 2023 and Ishida et al., 2009), but because we observed them on the reproductive parts of the flowers, we included them here, with the expectation of a negative association with burn severity, given their dependence on live trees as adults.

## 2 METHODS

### 2.1 Study area

**FIGURE 1:**
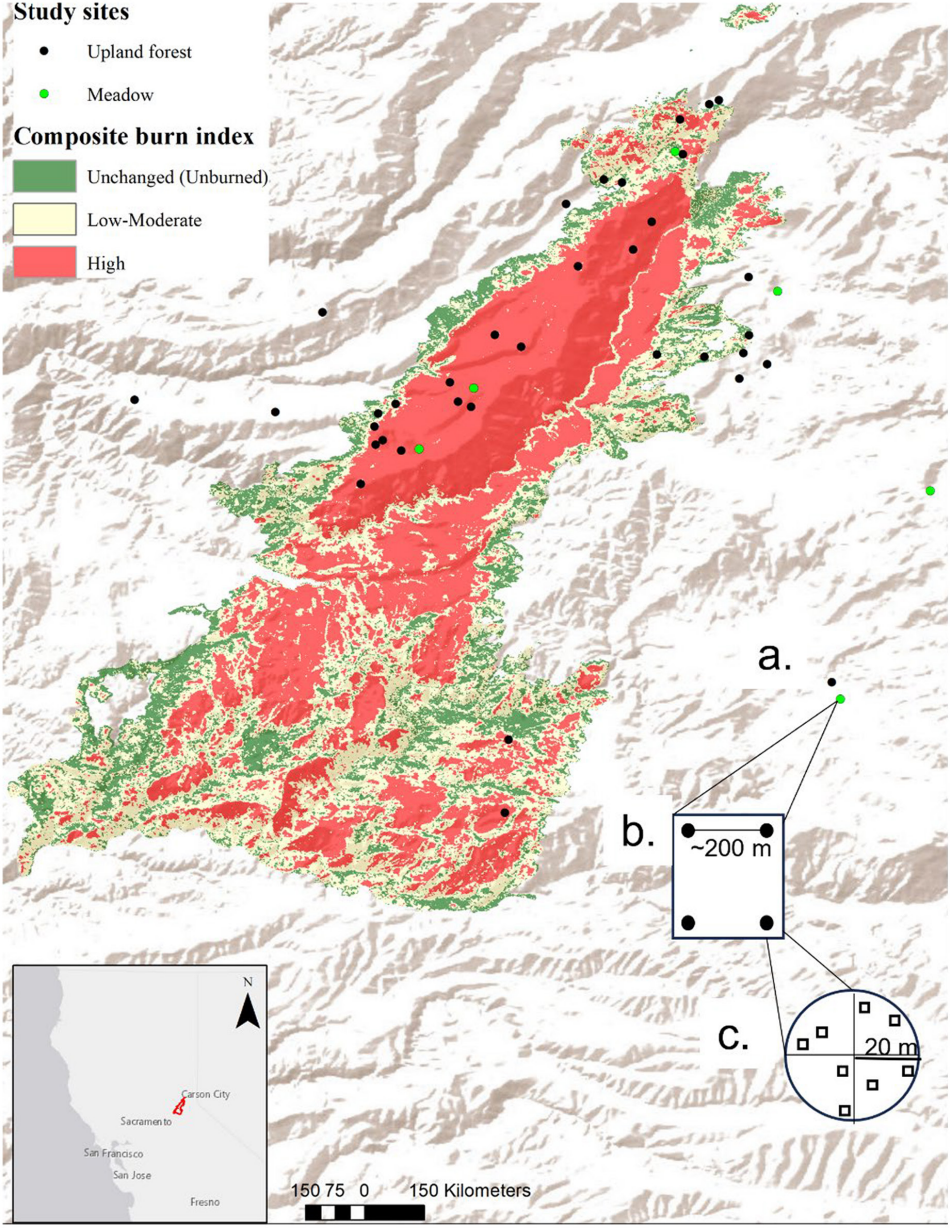
Map of the study area showing the region of the Sierra Nevada, California, where the King Fire burned in 2014; the inset shows the location of the King fire in California. (a) Green points are meadow, and black points are upland forest sites located in unburned, low‐moderate, and high‐severity burn classes. (b) Each site (square) consists of multiple 20‐m‐radius pollinator plots (circles) that were at least 100 m apart. (c) Pollinators were surveyed within each circular plot (circle), and plants in bloom were surveyed in eight randomly located 1‐m^2^ quadrats (squares) within each plot. Base layer sources: Esri, USGS, NOAA.

Our study area was located in and around the 2014 King Fire in the Eldorado National Forest, California (Figure 1). This region's climate is characterized by wet, cool winters with most precipitation falling as snow and dry, warm summers with little precipitation. Our study was restricted to upland and meadow communities (between ~1300 and 1800 m above sea level) to minimize the effects of elevation and related abiotic (e.g., precipitation) and biotic (e.g., vegetation communities) factors. The pre‐fire upland was largely composed of dense stands of relatively young white fir (*Abies concolor*), ponderosa pine (*Pinus ponderosa*), sugar pine (*Pinus lambertiana*), Douglas fir (*Pseudotsuga menziseii*), and incense cedar (*Calocedrus decurrens*). Pre‐fire meadows were dominated by grasses (Poaceae) and graminoids (Juncaceae and Cyperaceae), forbs, and small shrubs, with some conifer encroachment (McKelvey et al., 1996; Skinner & Chang, 1996). Given our focus on upland and meadows, we avoided sampling in areas classified as montane chaparral or riparian.

The Sierra Nevada mountains in California are home to a diverse community of diurnal pollinator species, including hummingbirds, butterflies, bees, and wasps (Hymenoptera: Apocrita), flies, and beetles that evolved with frequent low‐ to moderate‐severity fire (Bond & Keeley, 2005; Brook et al., 2008). Historically, fires in the mid‐elevation, mixed coniferous uplands of the Sierra Nevada burned every 5–25 years at low to moderate severity with some high‐severity patches (Beaty & Taylor, 2008; Collins & Stephens, 2010). Mid‐elevation meadows experienced severe fire every 200–300 years, typically following prolonged drought, with low‐severity fires occurring every 40 years (Caprio & Lineback, 2002; Ratliff, 1985). Prior to Euro‐American colonization in the mid‐1800s, the Nisenan, Washoe, and Miwok peoples managed the forest, woodlands, and meadows with low‐severity, frequent fires to stimulate the growth of understory plants that were important for food, fiber, medicine, or to attract game (Anderson & Moratto, 1996; Klimaszewski‐Patterson et al., 2018; Lake et al., 2017). The effect of Euro‐American settlement on the fire regime was twofold: first, it violently separated Indigenous people from the land they managed, and second, it enacted a forest management strategy that considered fire a destructive force to be excluded (Domínguez & Luoma, 2020; Hagmann et al., 2021; Kimmerer & Lake, 2001). The intensive logging, grazing, and fire suppression of the next century resulted in the buildup of dense, even‐aged stands and ladder fuels, which interact with contemporary urbanization, land use changes, and climate change to produce a fire regime characterized by less frequent but more severe wildfires (Dennison et al., 2014; Stephens et al., 2014; Williams, 2013).

The effects of the King fire were considered outside the natural range of variation for this fire regime (Safford & Stevens, 2017). The King fire was started on September 13, 2014, by an arsonist outside of Pollock Pines, California (38.782° N, 120.604° W). Fuels were abundant due to effective fire suppression that had excluded fire in the area for nearly 100 years (Department of Forestry and Fire Protection, 2018) and extremely dry due to a severe 3‐year drought, with low relative humidity and record high temperatures (Young et al., 2017). Over the next month, the King fire burned nearly 39,545 ha, about 50% at high severity (i.e., greater than 75% tree mortality, USDA Forest Service, 2014; Figure 1).

### 2.2 Sampling design

**FIGURE A1:**
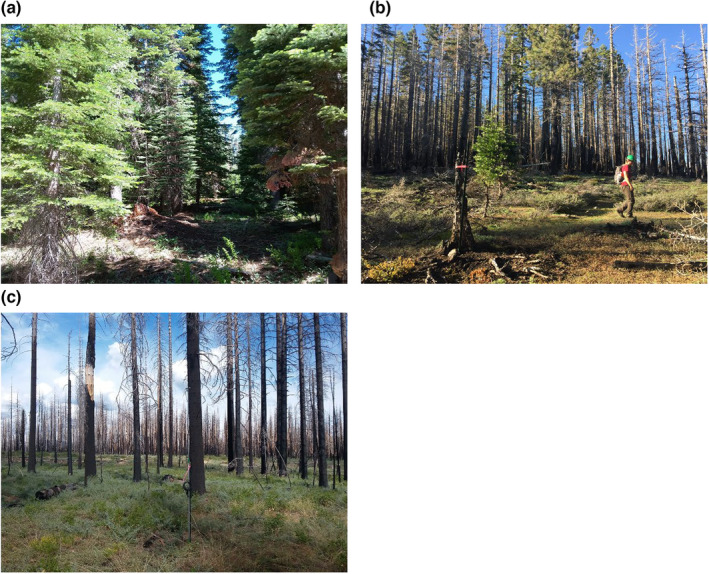
Photos taken at upland sites 3 years after the 2014 King Fire that were characterized as (a) unburned, (b) moderate‐severity, and (c) high‐severity burn classes.

Sampled sites were located in roughly homogenous areas (~200 m^2^) within a given burn severity class determined using the US Forest Service King fire RAVG (USDA Forest Service, 2014) composite burn index. The composite burn index is created by the Forest Service using both field and remote‐sensed data, with categories of unchanged (henceforth, unburned), low (surface fire with little mortality of dominant vegetation), moderate (mix of surface fire with little mortality and more severe fire with some mortality of the dominant vegetation), and high‐severity fire (dominant vegetation has high to complete mortality; Figure 1a). Low‐ and moderate‐ severity fires were limited within the fire perimeter, so we grouped them into one category of low to moderate severity (henceforth, moderate) to allow for adequate sampling. We excluded private lands, areas slated for post‐fire management (logging and other site preparation for tree planting), and areas that were inaccessible due to slope (>30%) or distance (>1 km) from roads. Sites were selected from the remaining area using ArcGIS^i^ 10.6 (ESRI, Redlands, California, USA) in unburned and high‐severity upland sites and unburned and burned meadow sites in 2016 and 2017, with moderate‐severity upland sites added in 2017. Ground‐truthing ensured that sites were assigned the appropriate burn severity category and habitat type (Figure A1).

In 2016, nine upland sites were established: six in upland habitat that burned at high severity and three in unburned habitat outside of the fire perimeter. All nine sites were visited three times following the spring snowmelt to account for some of the variation in phenology in the pollinator communities. In 2017, 27 new sites were established in uplands, with nine in each burn class (unburned, moderate severity, and high severity). In 2017, upland sites were only visited twice due to a truncated floral season that resulted from a cold, snowy spring, and to accommodate simultaneous sampling at moderate‐severity upland sites. We used a hierarchical sampling design, with multiple (3–5) 20‐m‐radius circular plots nested within sites of a particular burn‐habitat class (Figure 1b). Circular plots were separated by at least 100 m (average distance = 198 m) in either a linear or square orientation that best characterized the habitat of interest (Figure 1b). This resulted in a total of 151 unique upland plots on 36 sites over both years of sampling.

Meadow habitat was limited within the fire perimeter, and we sampled the same sites (but not necessarily the same plots) in both years of the study. Meadow sites were located within the fire perimeter (*n* = 3) or outside the fire perimeter (*n* = 3) and were visited three times per season in both years of the study. In 2016, each meadow site had five plots, for a total of 30 meadow plots. This was reduced in 2017 to three or four plots per site for a total of 22 meadow plots due to the logistic constraints outlined above (Table A1). To ensure that differences in pollinator abundance were due to ecological differences rather than sampling error, we used iNEXT (Hsieh et al., 2020) to evaluate sample completeness and found that coverage was high, particularly for meadows, and similar across habitats (Table A2).

Plots were visited from June through September during daylight hours when weather conditions supported insect activity: temperatures were ≥10°C, wind speeds were below 11.2 ms^−1^, with no precipitation (Loffland et al., 2017). In each plot, we surveyed pollinators and flowering plants with open inflorescences. Pollinators that were targeted using our survey methods included bumble bees, butterflies, and hummingbirds in taxa‐specific surveys in 2016. However, to more effectively survey the pollinator community and the floral resources utilized, we shifted our focus in 2017 to record all pollinators in timed surveys as they visited flowers within the plots (flower‐visitor surveys).

### 2.3 Flower‐visitor surveys

We sampled *Bombus* species and other insect pollinators separately, using plot size and sampling periods following other studies to allow comparison of our study with *Bombus* populations in other Sierra Nevada fires (Cole et al., 2020; Loffland et al., 2017). Two observers sampled each 20‐m‐radius plot (Figure 1c) for all *Bombus* species in two consecutive 16‐min surveys (2016 and 2017) and all other insects in one separate 16‐min survey (2017 only). These times reflect the minimum amount needed for a surveyor to scan the entire plot, maximizing capture while minimizing movement of pollinators in and out of the plot (i.e., to ensure population closure). Although we used two consecutive sampling periods to survey *Bombus* to account for detectability using a removal model (Farnsworth et al., 2002), we ultimately did not observe a decline in detections from the first to the second period, thus we pooled data from the two sampling periods.

Each individual was captured with a 381‐mm‐sized insect net, placed in a vial, and held in a cooler until the end of the survey period. Once the survey was complete, individuals were either identified by species and released or collected for later identification by species or morphospecies using published keys or expert opinion (Triplehorn & Johnson, 2005; UC Davis Bohart Museum). All specimens were mounted and vouchered in the UC Davis Bohart Museum of Entomology.

We also recorded the plant species visited and whether the pollinator was captured in the air or on another substrate. Although visitation does not necessarily correspond to pollination, the two are highly correlated (Alarcón, 2010), and we refer to flower visitors as pollinators for simplicity.

### 2.4 Butterfly surveys

In 2016, we conducted 5‐min point counts for butterflies at each plot on each visit. An observer stood at the plot center and attempted to count every individual that entered the plot during this time (Henry et al., 2015; Lang et al., 2019). Five‐minute point count surveys for butterflies provide unbiased density estimates, particularly in dense or heterogeneous habitats (Henry et al., 2015; Van Swaay et al., 2012). Butterflies were identified by family or lower taxonomic classification on the wing or captured in a 381‐mm insect net for identification in the hand as needed (surveys were paused while capturing and identifying species). In 2017, we dropped the point counts because butterflies were included in the flower‐visitor surveys. Unfortunately, they were too rare to be analyzed, so we only present results from 2016.

### 2.5 Hummingbird surveys

In 2016, hummingbirds were sampled using a mix of passive and broadcast surveys to better estimate detection probability (Loffland et al., 2011; Saracco et al., 2011). Broadcast surveys are often used to detect rare or elusive species (Saracco et al., 2011), and we hypothesized that the territorial nature of hummingbirds suited them for this sampling method. During each survey, we conducted a 5‐min passive survey immediately followed by a 6‐min broadcast survey (30 s of broadcasting, then 90 s of observing, repeated three times) for each species (*Calypte anna*, *Selasphorus rufus*, and *S. calliope*) at each plot on each visit. Each 30‐s recording consisted of wing and tail buzz sounds, dive display calls, and chip notes (Macaulay Library of Natural Sounds, Cornell Laboratory of Ornithology, https://macaulaylibrary.org). If a hummingbird was detected, the detection was noted as occurring during one of the sampling intervals (1 passive interval, 3 active intervals per species). Because many detections of hummingbirds were incidental while collecting data on other taxa, we dropped the broadcast surveys in 2017. Hummingbirds were included in the flower‐visitor surveys, and in addition, observations of hummingbirds were collected opportunistically at each visit while sampling for other species.

### 2.6 Floral resources

We estimated the abundance and richness of flowering plants in bloom in eight 1‐m^2^ quadrats in each plot (Figure 1c). We chose to only include plants with open flowers because they best represented the food resources of nectar and pollen available to pollinators during the survey period. Plots were divided into quarters using transects, and two quadrats were randomly placed in each quarter. In each quadrat, we identified every plant in flower by species following the Jepson manual (Baldwin et al., 2012) and counted all inflorescences with open flowers. Because pollinators visited some 147 recorded plant species rarely (*n* = 45 with <5 visits) or never (*n* = 63), we only included a subset of the total plant species that were visited frequently (*n* = 30 with ≥10 visits) in our analysis (Table A3). Floral abundance was defined as the sum of inflorescences with open flowers on frequently visited species in each plot for each visit. Floral richness was the number of frequently visited species with open flowers found in all quadrats of a plot on each visit.

### 2.7 Analysis

To investigate how burn severity, habitat type, and floral resources affect pollinator abundance, we created hierarchical generalized linear mixed models (GLMMs) for each of the following taxonomic groups: bumblebees, butterflies, other insects, and hummingbirds, where site was included as a random effect to account for the nested sampling design and repeated sampling (e.g., Gelman & Hill, 2006). We modeled the abundance of pollinator groups separately to account for differences in sampling effort/method and number of individuals detected. Abundance was calculated for each visit, site, and year (when applicable) as the total number of detections of a given species.

Bumblebees and other insects were each modeled with hierarchical multi‐species abundance models, where multiple species are nested within the larger community. Species‐specific parameters come from a hyperdistribution that is shared by all species and described by hyperparameters (Dorazio et al., 2006; Dorazio & Royle, 2005). Hierarchical multi‐species abundance models share information within the community, allowing us to model relatively rare species, but they require a sufficient number of species (typically 6 or more) per community (Zipkin et al., 2010). To improve model fit and convergence, we only included species observed in at least three plots in community models (see below for model specification). We did not have enough taxonomic resolution to use community models on butterflies or hummingbirds. Instead, we created GLMMs for each butterfly family and one GLMM for all hummingbirds. Abundances of all taxa were modeled with the following covariates: burn severity and habitat type (upland or meadow) and an interaction term, year (only for *Bombus* and hummingbirds, which were sampled in both years), floral abundance and richness, elevation, and days since snowmelt.

Although sites were selected using a categorical assessment of burn severity, we modeled abundance with a continuous value of burn severity, the Relative differenced Normalized Burn Ratio (RdNBR), due to its greater accuracy in high severity, heterogeneous landscapes and the finer resolution possible with this metric (Miller & Thode, 2007). RdNBR is derived from the Normalized Burn Ratio, a vegetation index that used LiDAR data regressed with field data to detect differences in live unburned vegetation from dead wood, moisture content, and mineral and soil conditions; it was calculated by the US Forest Service at 30 x 30m resolution across the King Fire (RAVG, USDA Forest Service, 2014). We assigned each plot an RdNBR value based on the pixel it fell within. In upland habitat, we also included a quadratic term for burn severity to account for the non‐linear response that has been observed for understory plants in this region (Richter et al., 2019). This was not evaluated for meadows because intermediate RdNBR values were not well represented in meadow habitat.

Because we were interested in how the response of pollinators to burn severity may be mediated by floral resources, we included two metrics to account for the importance of these resources to pollinators: floral abundance and richness. Because the floral resource variables were correlated with the habitat type (Figure A1, Table A4), we standardized the floral abundance and richness with the mean and standard deviation of their respective habitats. Thus, the baseline difference in floral resources among meadow and upland habitats is incorporated into the categorical habitat covariate, and the floral abundance and richness covariates describe the deviation from the habitat‐level mean.

Elevation and days since snowmelt were included in all models to explain additional variation in abundance due to unmeasured environmental factors likely correlated with these variables (e.g., temperature) and to improve model fit. Elevation was derived from digital elevation models in ArcGIS. We estimated snowmelt dates for each year (June 6, 2016 and June 18, 2017) using data from the Greek Store (GKS) and Robb's Saddle (RBB) weather stations located close to (~10 km) and at similar elevations (~1700 m asl) as the study area (California Department of Water Resources, 2018). The days since snowmelt were the difference between the date of each visit and the regional date for snowmelt for each year, scaled to have a mean of 0 and a standard deviation of 1. Year was included in models for taxa surveyed in multiple years (bumblebees and hummingbirds) to account for annual variation in pollinator communities and changes in sampling protocols. We used negative binomial models to account for overdispersion in count data.

Seven *Bombus* species were included in one multi‐species (community) abundance model, and the other 30 insect visitors in another. Abundance *N*_*ijk*_ of species *i* = 1,2,…,*n* at each of the *j =* 1, 2,…,*J* plots at each visit *k* = 1, 2,…,*K* was modeled as a negative binomial random variable with species‐ and plot‐specific mean ($\lambda_{\mathrm{ijk}}$) and a common dispersion parameter *r*:

$$N_{\mathrm{ijk}}\sim \text{Negative binomial}\left(\lambda_{\mathrm{ijk}},r\right)$$

$$\begin{array}{c} \log\;\left(\lambda_{\mathrm{ijk}}\right)=\beta 0_{i}+\beta 1_{i}{\text{Snowmelt}}_{\mathrm{jk}}+\beta 2_{i}{\text{Richness}}_{\mathrm{jk}}\\ \;+\beta 3_{i}{\text{Inflor}}_{\mathrm{jk}}+\beta 4_{i}{\text{Elevation}}_{j}+\beta 5_{i}{\text{Year}}_{\mathrm{jk}}\\ \;+\beta 6_{i}{\text{Burn}}_{j}+\beta 7_{i\left[\text{Habitat}=\text{upland}\right]}{\text{Burn}}_{j}^{2}\\ \;+\beta 8_{i}{\text{Habitat}}_{j}{\text{Burn}}_{j}+\varepsilon_{\text{site}\left[j\right]} \end{array}$$

Here, $\beta 0_{i}\sim \text{Normal}\left(\mu .\beta 0,\;\sigma .\beta 0\right)$ and analogous for all other coefficients; μ.β0 is the community mean coefficient and σ.β0 is the community standard deviation, which describe the variation of species‐level coefficients about the community mean. Snowmelt is the days since snow melt in days, Richness is floral richness, Inflor is floral abundance, and Elevation is elevation in meters; and ε_i_ is a normally distributed random effect of site, accounting for both the study design (plots nested within sites) and repeated sampling (e.g., Gelman & Hill, 2006). Year (reference = 2016) was included as a fixed effect in the *Bombus* model to account for interannual variation. The quadratic term for burn severity was only included for upland habitat; that is, β7 was fixed at 0 for meadow habitat. All continuous variables were centered and standardized.

We modeled abundance separately for each family of butterfly and for all hummingbirds combined, using single‐species negative binomial models with the same general model structure (covariates and random effect) as described above. Year and the quadratic burn severity term were not included in the butterfly model because they were only surveyed in 2016 at unburned and high‐severity plots.

We implemented all models in a Bayesian framework using the software JAGS version 4.3.0 (Plummer, 2003), accessed through the jagsUI package 1.5.1 (Kellner, 2021), in R version 3.4.4 (R Core Team, 2023). All parameters were assigned vaguely informative priors. The posterior distributions were sampled using three Markov Chain Monte Carlo (MCMC) chains, each with 100,000 samples and a burn‐in of 50,000 samples. The convergence of MCMC chains was evaluated using traceplots and the Gelman‐Rubin statistic (where $\hat{R}$ ≤ 1.1 is considered convergence; Gelman et al., 2004). We report posterior means, standard deviations (SD), and the 2.5th and 97.5th percentiles as the 95% Bayesian credible interval (BCI) for each parameter; we considered coefficients whose 95% BCI did not overlap zero as significant. We calculated Bayesian *p*‐values for the species and community abundances to evaluate goodness‐of‐fit (Bayesian *p*‐value between .1 and .9 indicate fit; Gelman et al., 1996; Kéry & Royle, 2020; Appendix A). All chains converged, and all models fit their respective data appropriately (Tables B2, B3, B4, B5).

## 3 RESULTS

### 3.1 Flower visitors

#### 3.1.1 *Bombus* species

**FIGURE 2:**
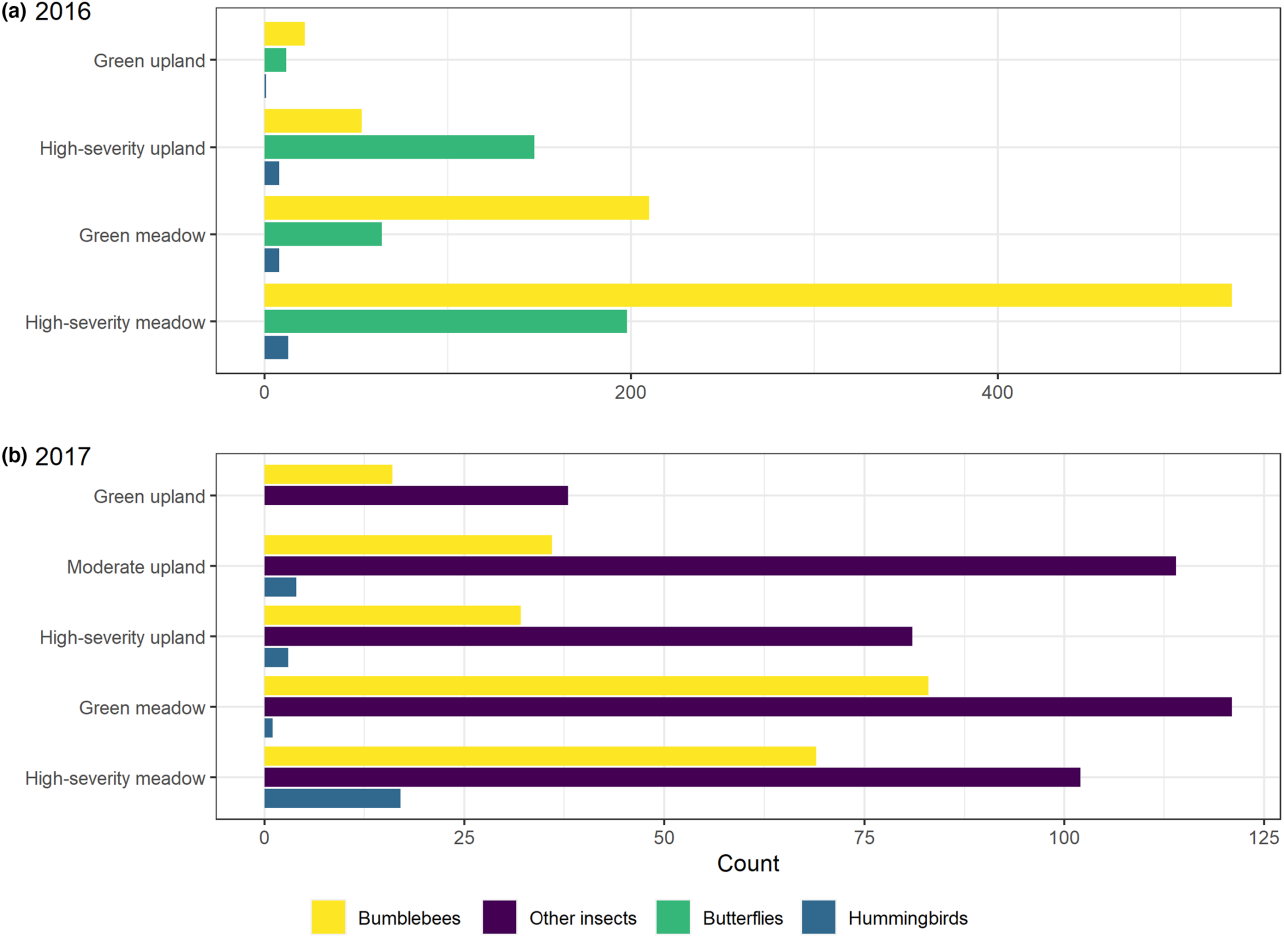
Pollinator abundance by year, burn‐habitat class, and taxon, during surveys in the Sierra Nevada, California, 2 and 3 years after the 2014 King Fire. Moderate‐severity habitat was only sampled in 2017. Butterflies were only sampled in 2016, and other insects (including bees, wasps, flies, true bugs, and beetles) were only sampled in 2017. Note the difference in scales on the x‐axis; different taxa were sampled with different methodologies, precluding among‐taxon comparison of abundance (see main text for details).

In 2016, we captured 812 bumblebees from nine species; eight individuals escaped prior to identification. In 2017, we captured 233 *Bombus* individuals from nine species; two individuals escaped prior to identification (Figure 2). Bumblebees that were not identified as species were dropped from further analysis (Table A5). *B. vosnesenskii* was the most commonly encountered species in all burn severity–habitat combinations in both years of sampling. Across both years, 1019 observations of seven species of *Bombus* were included in the community model (Tables B1 and B2).

**FIGURE 3:**
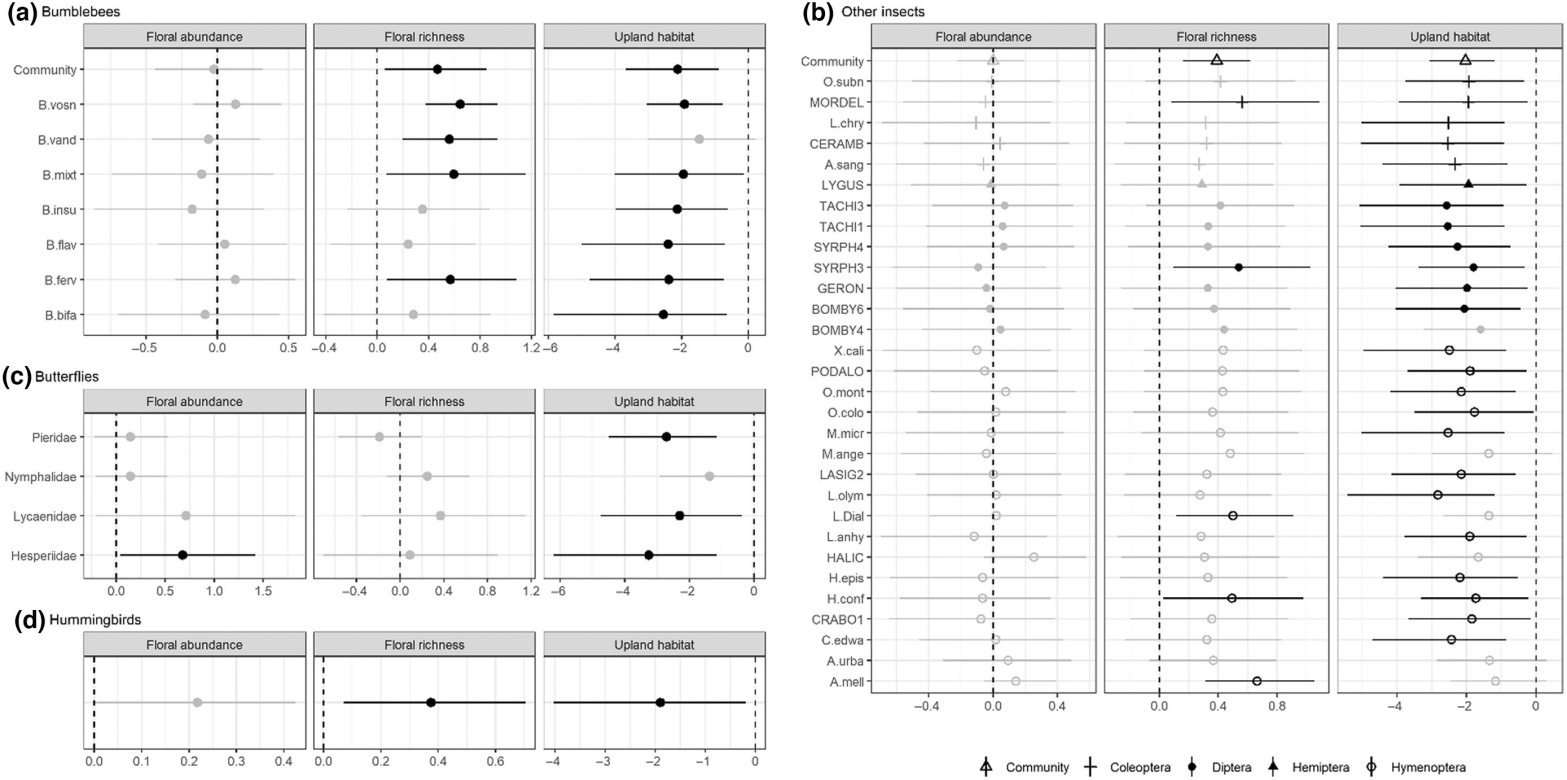
Factors influencing the abundance of pollinator taxa in meadow and upland habitat in the Sierra Nevada, California, 2 and 3 years after the 2014 King Fire, estimated using negative binomial generalized linear mixed models (single‐species models for butterflies and hummingbirds, multi‐species models for bumblebees and other insects). Covariate coefficients for habitat type and cover and richness of blooming plant species visited at least 10 times by pollinators for (a) the bumblebee community on average and individual species, (b) the community of other flower‐visiting insects on average and individual species, (c) butterfly families, and (d) hummingbirds. Negative coefficients represent a decline in abundance; positive coefficients indicate an increase in abundance with increasing covariate value. Coefficients with significant effects (i.e., 95% Bayesian credible intervals do not overlap 0) are indicated by the darker shaded points. Species codes are in Table A5.

**FIGURE 4:**
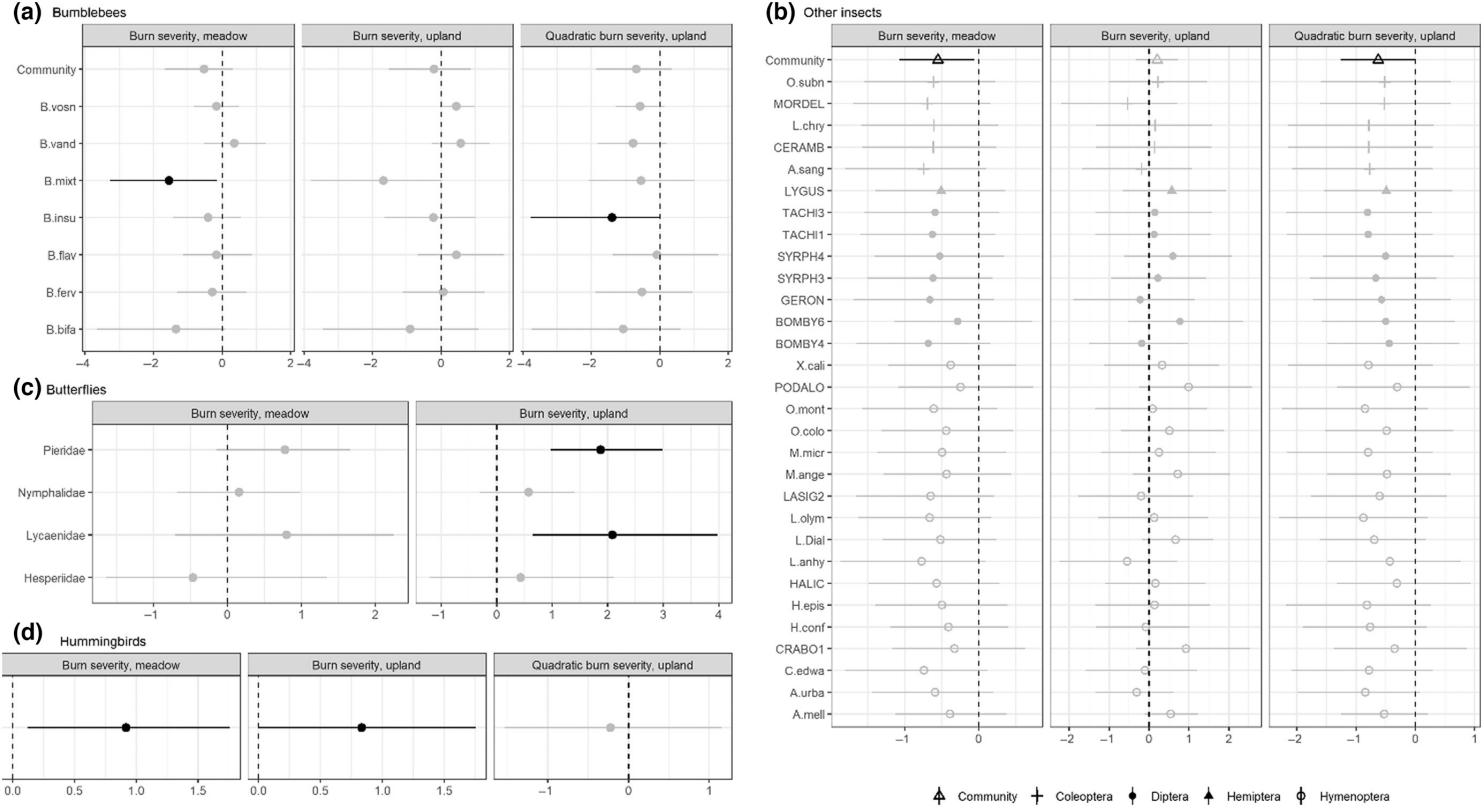
Factors influencing abundance of pollinator taxa in meadow and upland in the Sierra Nevada, California, 2 and 3 years after the 2014 King Fire, estimated with negative binomial generalized linear mixed models (single‐species models for butterflies and hummingbirds, multi‐species models for bumblebees and other insects). Covariate coefficients for the interaction of burn severity and habitat, and the quadratic term for burn severity in upland habitat only for (a) the bumblebee community on average and individual species, (b) the community of other insects on average and individual species, (c) butterfly families (not sampled in moderate‐severity habitat, so no quadratic burn effect), and (d) hummingbirds. Negative coefficients represent a decline in abundance; positive coefficients indicate an increase in abundance with increasing burn severity. Coefficients with significant effects (i.e., 95% Bayesian credible intervals do not overlap 0) are indicated by the darker shaded points. Species codes are in Table A5.

Bumblebee abundance was significantly higher in meadows than upland habitat for the overall community (hyperparameter) and for all individual species except *B. vandykei* (Figure 3a). Bumblebee community abundance was not associated with burn severity in meadow (linear) or upland habitat (linear, quadratic; Figure 4a). However, at the species level, bumblebee abundance in meadows tended to decrease with increasing burn severity, although the effect was only significant for *B. mixtus*. In upland habitat, the effect of burn severity varied, but abundance tended to be highest for all species at moderate RdNBR levels, with the effect only reaching significance for *B. insularis* (quadradic). However, bumblebee abundance was positively associated with floral richness for all species, and the relationship was significant at the community level and for *B. vosnesenskii*, *B. vandykei*, *B. mixtus*, and *B. fernalde* (Figure 3a). Surprisingly, the effect of floral abundance was close to zero at the community level and not significant for any species (Figure 3a). There were no significant effects of time since snowmelt or elevation, but *B. vosnesenskii*, *B. mixtus*, and *B. bifarius* were significantly more abundant in 2016 and *B. insularis* was significantly more abundant in 2017 (Table B2).

#### 3.1.2 Other insects

In 2017, we observed 681 individuals representing 132 species or morphospecies from six orders of insects (Figure 2, Table A5). Most individuals were of the order Hymenoptera, family Apidae (*n* = 381). After omitting species that were detected in fewer than three plots, we included 286 individuals of 30 species from five orders in the community model for non‐*Bombus* insects.

The insect community was significantly more abundant in meadow habitat (Figure 3b, Tables B1, B3), with the effect of upland habitat negative for all species and reaching the level of significance for 24 species. In meadows, the abundance of the insect community decreased significantly with burn severity, and although all species‐level parameter estimates were negative, none were significant. In contrast, in upland habitat, species‐level abundances tended to increase with burn severity, with the highest abundances at moderate levels of burn severity, but the effect only reached significance at the community level (quadratic; Figure 4b). Contrary to our predictions, the abundance of beetles, flies, and true bugs were not significantly associated with upland habitat or burn severity. The species‐level response to floral richness was positive for all species (Figure 3b) and significant for one beetle (*Mordella* species), one fly (Syrphidae morphospecies), and three bees (*Lasioglossum* (Subgenus: dialictus)*, Halictus confusus*, and *Apis mellifera*), and we found a positive and significant effect of floral richness on the community‐level insect abundance (Figure 3b). The abundance of insects (community or species level) was not influenced by floral abundance. Elevation did not have a significant effect, but the abundance of 10 species decreased significantly with the increasing number of days since snowmelt (Table B2).

### 3.2 Butterflies

In 2016, we observed 421 individuals from five butterfly families (Figure 2, Table A5). We included 419 butterfly observations from four families in family‐specific abundance models. We observed more butterfly pollinators in meadow habitat than upland habitat, and this relationship was significant for three out of four families (Pieridae, Lycaenidae, and Hesperiidae) and marginally significant for the fourth (Nymphalidae; Figure 3c, Tables B1, B4). There was no significant response of butterfly abundance to burn severity in meadows, but two families, Pieridae and Lycaenidae, increased significantly with increasing burn severity in uplands (Figure 4c). Only Hesperiidae was significantly associated with increasing floral abundance, although we did observe a nonsignificant positive trend for all families (Figure 3c). Similarly, although no families were significantly associated with floral richness, three out of four families (Nymphalidae, Lycaenidae, and Hesperiidae) tended toward higher abundance with increasing richness. Nymphalidae and Lycaenidae both decreased significantly with increasing elevation, while Pieridae and Herperiidae both decreased significantly with increasing number of days since snowmelt (Table B4).

### 3.3 Hummingbirds

In 2016, we detected 30 hummingbirds in both broadcast surveys and incidental observations during plot surveys (Figure 2; Figure A1, Table A5). In 2017, we observed 25 hummingbirds incidentally at plots. We included all 55 observations in our hummingbird abundance model.

We found that the effect of burn severity was significantly positive for hummingbird abundance in both meadow and upland habitat, but there was no evidence for a quadratic effect of burn severity in upland habitat (Figure 4d; Tables B1, B5). As we predicted, hummingbird abundance increased with increasing floral richness and abundance (Figure 3d), and they were more abundant in meadows than uplands. Hummingbirds decreased significantly with the increasing number of days since snowmelt but were not affected by elevation or year (Table B5).

## 4 DISCUSSION

We found that pollinators were more abundant in meadow habitat, but for some taxa, abundance tended to decrease at the high burn severities observed in the King fire and other large wildfires of Mediterranean climates (Carbone et al., 2019, Lazarina et al., 2019). In upland habitat, our results supported previous studies that found pollinators to be positively associated with upland habitat that burned at moderate severities, suggesting that both unburned and highly burned upland forests supported lower abundances of pollinators (Lazarina et al., 2019). These patterns suggest that an increase in the frequency and size of high‐severity fires will have negative consequences for pollinators. Management efforts to increase the area of meadows by reducing conifer encroachment (Boisramé et al., 2019; Kirkland et al., 2023) and increasing the amount of fire at low‐ to moderate‐severity in this fire‐prone system (Cansler et al., 2022; North et al., 2021; Young et al., 2020) would be beneficial to pollinators (Lazarina et al., 2019; Ponisio et al., 2016). Increasing the proportion of low‐ to moderate‐severity fire on the landscape is likely also beneficial for soils (Certini, 2005), plants (Pourreza et al., 2014; Richter et al., 2019), trees (Dunn et al., 2020), and other wildlife (Furnas et al., 2022; Kramer et al., 2021; Taillie et al., 2018).

**FIGURE A2:**
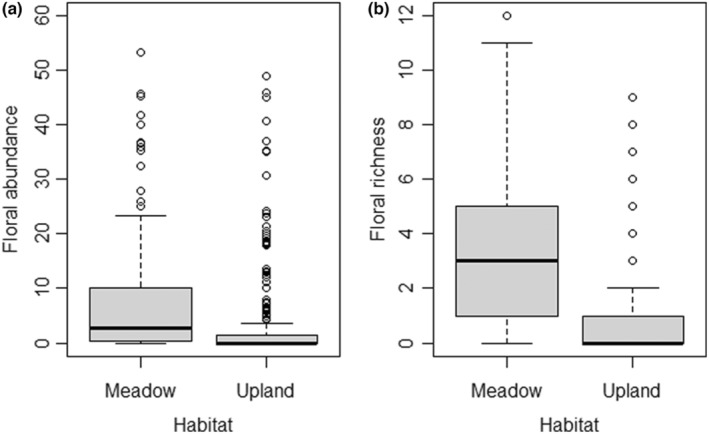
Correlation between meadow and upland habitats and a. floral abundance and b. floral richness in the Sierra Nevada, California, 2 and 3 years after the King Fire.

We expected most pollinator taxa to have higher abundances in meadow than upland habitat regardless of burn severity, with the exception of beetles, flies, and bugs that may rely on upland habitat in larval stages. In fact, we found that all taxa were positively associated with meadow habitat, and nearly all associations were significant. Meadows provide an abundance of floral resources, with higher average diversity and cover than upland habitats (Figure A2), making these resources easier to find in meadows (Dávalos & Blossey, 2011). Additionally, pollinators may be particularly attracted to meadow habitats because they provide abundant host plants (van Nouhuys & Hanski, 2005), opportunities for mud puddling (Downes, 1973), increased visibility for mate‐seeking (Dennis & Shreeve, 1988), courtship displays (Mikula et al., 2022), and higher light levels for flight (Ross et al., 2005). For example, caterpillars of many butterfly species are dependent on particular host species or genera, which are often associated with or limited to mesic habitats (Dennis & Shreeve, 1988; Fleishman, 2000). Male butterflies are known to guard host plants when searching for mates, which may increase their incidence in meadows (Dennis & Shreeve, 1988; Fleishman, 2000). Because meadows in the Sierra Nevada and many other regions are located within a forest matrix, there is local heterogeneity that may provide complementary resources for pollinators (Diaz‐Forero et al., 2013; Liivamägi et al., 2014; Martins et al., 2015). For example, meadow‐adjacent forest may provide the dead and decaying wood required by flower‐feeding beetles for egg‐laying and larval development at larger spatial scales (Horak, 2014; Rubene et al., 2017). Similar landscape‐scale resource acquisition may occur in other taxa, such as flies, true bugs, and butterflies; further research into larval abundance may reveal responses to habitat heterogeneity or shifts in preferences over time.

The propensity for pollinator species to be more abundant in meadows is particularly concerning given their response to high‐severity fire in this habitat, with consistent (though mostly insignificant) declines in abundance with increasing burn severity. High‐severity fire in meadows tends to be directly related to prolonged drought, which results in a continuous layer of matted surface fuels and a thick layer of decomposed organic soil (DeBenedetti & Parsons, 1979; Kirkland et al., 2023). These extreme conditions often result in smoldering fires that burn hotter with longer residence times and may effectively sterilize the soil (Rein et al., 2008). Post‐fire, meadow vegetation tends to recover quickly, but there is often a shift from perennial to annual species (Tarbill, 2022), which tend to produce significantly less pollen and nectar (Potts, Vulliamy, Dafni, Ne'eman, O'toole, et al., 2003; Potts, Vulliamy, Dafni, Ne'eman, & Willmer, 2003; Hicks et al., 2016). Both the direct effects of fire on pollinator mortality and the indirect effects through plant community composition may explain the negative relationships between abundance and burn severity we observed in meadows.

Meadows, including burned meadows, provide highly concentrated, diverse, and somewhat permanent floral resources, and therefore may be more reliable than the patchy and often ephemeral floral resources of upland habitat (Clark & Russell, 2020; Gass, 1979; Hatfield & LeBuhn, 2007). The hummingbirds were the only pollinator to be positively and significantly associated with burn severity in meadows. Hummingbirds often exhibit site fidelity to habitat used during breeding or migration (Calder et al., 1983), and the use of burned meadows may be maladaptive (Merkle et al., 2022; Shochat et al., 2005). Alternatively, hummingbird abundance may be positively associated with burn severity in meadows if fire increases the availability of resources when they are migrating through or breeding (McKinney et al., 2012; Russell et al., 1994). Hummingbird abundance declined significantly as the season progressed, suggesting that they target habitats with relatively early‐blooming flowers. Burned meadows may have higher floral diversity earlier in the season relative to unburned meadows (Tarbill, 2022), suggesting that fire‐induced resource pulses in meadows may align with the needs of hummingbirds. Burned meadows dominated by annual floral resources may be adequate for hummingbirds that tend to utilize flowers with a lower nectar concentration than bees and butterflies (Nicolson, 2022). More research into the reproductive success or long‐term survival of these species in burned and unburned meadows is needed to understand this relationship.

The increase in the abundance of pollinators at moderate levels of burn severity in upland forest agrees with recent pollinator studies in systems with similar disturbance regimes (Lazarina et al., 2019) and is attributed to increased habitat heterogeneity and the “pyrodiversity begets biodiversity” hypothesis (Martin & Sapsis, 1992; Ponisio et al., 2016; Ulyshen et al., 2022). Pyrodiversity accounts for characteristics of a burned area that may vary in time, severity, and seasonality, resulting in a spatial representation of diverse fire histories. Fire in our study area was very effectively suppressed over the past century; thus, there was no variation in fire history, just spatial variation in burn severity. This temporal homogeneity may explain why we did not observe a significant relationship with moderate‐severity fire at the species level, suggesting that variation in fire history may be important in structuring pollinator communities in fire‐prone systems (Ponisio et al., 2016; Ulyshen et al., 2022).

Some species or taxa responded to the fire in surprising ways. For example, two bumblebee species responded negatively to burn severity in both meadows and uplands: *Bombus mixtus* and *B. bifarius*. In contrast to other bumblebees, these species tend to nest on the surface or aboveground rather than in burrows (Hobbs, 1967; Foster, 1992; Wray & Elle, 2015), which may increase vulnerability to wildfire at any severity (Cane & Neff, 2011). On the other hand, two butterfly families responded positively to burn severity in upland habitat. However, due to data limitations, we were unable to test if the abundance of these families peaked with moderate‐severity fire. Other studies have found a variety of responses of butterflies to fire, dependent on burn severity, habitat type, and fire frequency (Mason Jr et al., 2021). Furthermore, butterflies, even within families, are highly variable in host plant specificity (Dyer et al., 2007), and this will likely affect butterfly response to fire at the species level (Gaigher et al., 2019; Huntzinger, 2003). For example, fire may improve habitat for monarch butterflies (Nymphalidae: *Danaus plexippus*) by increasing the density of fire‐following *Asclepias* host species (Baum & Sharber, 2012; Ricono et al., 2018).

Floral richness was positively associated with nearly all pollinator communities and taxa (although not always significant), and the effect of floral richness was strongest (i.e., highest coefficient values) for bumblebees and the European honeybee. Multiple flowering plant species that bloom sequentially may be necessary to support bumblebees and honeybees that rely upon pollen and nectar in all active life stages, with colonies to support over an entire season (and beyond for honeybees storing honey for winter; Aldridge et al., 2011; Hemberger et al., 2022). We expected a weaker response from wasp species, but we found crabronid and *Podalonia* wasps were similar to other Hymenoptera species. Adult crabronid and *Podalonia* wasps are completely dependent on nectar for nutrition, explaining their association with floral resources. Perhaps the floral resources also attract the prey species they rely upon to provision their young. Increasing floral richness may positively impact pollinators by providing pollen and nectar resources that vary temporally and spatially, providing reliable food resources for long‐lived, early, and late emerging species, as well as multiple generations of social bees (Ebeling et al., 2008; Kaluza et al., 2018; Ogilvie & Forrest, 2017; Roulston & Goodell, 2011). High floral diversity may result in more visitors due to increased attraction of large mixed species displays (Ghazoul, 2006; Vaca‐Uribe et al., 2021) or reduced competition among pollinators (Brosi et al., 2017; Kaluza et al., 2017). High floral diversity may also increase the likelihood of specialist pollinators finding their preferred host due to sampling effects (Loreau et al., 2001).

Surprisingly, most species of pollinators were not associated with the abundance of open flowers. For non‐bee species, this may be explained by the reliance of larval stages on non‐floral resources, a pattern that has been observed for flies (Robinson et al., 2018), beetles (O'Neill et al., 2008), and butterflies (Woodcock et al., 2012) in other systems. Floral abundance may not affect the abundance of bees and other pollinators if there is a mismatch in the flower and pollinator morphology (Klumpers et al., 2019; Stang et al., 2009). Many plants with densely packed inflorescences have individual flowers that may be inaccessible or inefficient for larger pollinators to handle (Klumpers et al., 2019; Stang et al., 2009).

The pollinators of the mixed‐conifer forests of the Sierra Nevada evolved under a disturbance‐prone system that may have filtered out species that are intolerant of frequent environmental change (Kelt et al., 2017), resulting in a community that is resilient to frequent, albeit moderate‐severity fire. In fact, pollinators in this and other fire‐prone regions often benefit from moderate‐severity fire (Lazarina et al., 2019; Ponisio et al., 2016; Rodríguez & Kouki, 2017), even when embedded within large fires that burn at high severity. A related study found that the abundance and diversity of floral resources are higher in high‐severity upland habitat compared to unburned upland (Tarbill, 2022), suggesting that even high‐severity fire may create resources for pollinators that are lacking in unburned forest. This is reassuring, given that much of the Sierra Nevada is overdue to burn (North et al., 2012) and fire effects are likely to be severe (Cassell et al., 2019; Collins, 2014). Our study shows that pollinators can survive or (re)colonize high‐severity, large‐scale fires; however, their ability to do so will depend on landscape connectivity (Adedoja et al., 2019; Brown et al., 2017; Carbone et al., 2019), post‐fire management (Galbraith et al., 2019b; Heil & Burkle, 2018), and life history traits (Enright et al., 2014; Peralta et al., 2017; Williams et al., 2010). Globally, nearly 90% of wild flowering plants benefit from pollination services (Ollerton et al., 2011); pollinator abundance is associated with increased seed set and reduced pollen limitation in flowering plants (Cusser et al., 2016; Steffan‐Dewenter & Tscharntke, 1999; Thomson, 2019). Therefore, identifying the drivers that influence the abundance of pollinators, particularly after disturbances that are outside the historical range of variability, is critical to supporting pollination services under global change.

## AUTHOR CONTRIBUTIONS

**Gina Tarbill:** Conceptualization (equal); data curation (equal); formal analysis (equal); funding acquisition (equal); investigation (equal); methodology (equal); project administration (equal); writing – original draft (lead); writing – review and editing (equal). **Angela White:** Conceptualization (equal); data curation (equal); formal analysis (equal); funding acquisition (equal); investigation (equal); methodology (equal); project administration (equal); writing – original draft (supporting); writing – review and editing (equal). **Rahel Sollmann:** Conceptualization (equal); data curation (equal); formal analysis (equal); funding acquisition (equal); investigation (equal); methodology (equal); project administration (equal); writing – original draft (supporting); writing – review and editing (equal).

## CONFLICT OF INTEREST STATEMENT

We have no conflicts of interest to disclose.

## Data Availability

Data and code will be available at the USDA Forest Service Research Data Archive: https://www.fs.usda.gov/rds/archive.

## Endnote

^i^
The use of trade or firm names in this publication is for reader information and does not imply endorsement by the U.S. Department of Agriculture of any product or service.

## APPENDIX A

**TABLE A1:** Number of sites with plots in parenthesis surveyed in each year and burn‐habitat class.

	Green		Moderate	High	
	Meadow	Upland	Upland	Meadow	Upland
2016	3 (15)	3 (15)	NA	3 (15)	6 (30)
2017	3 (11)	9 (36)	9(36)	3 (11)	9 (36)
Total	26	51	36	26	66

**TABLE A2:** Number of species observed and estimated sampling coverage for each treatment.

Treatment	Observed species richness	Sampling coverage (%)
Green Meadow	57	81
Green Upland	28	69
High Meadow	58	80
High Upland	35	79
Moderate Upland	48	81

**TABLE A3:** Species or morphospecies of plants that had at least 10 pollinator visits in the Sierra Nevada, CA, 2 and 3 years after the 2014 King Fire.

Scientific name	Family	Native	Pollinator visits
*Erigeron* species	Asteraceae	Native	26
*Asyneuma prenanthoides*	Campanulaceae	Native	43
*Bistorta bistortoides*	Polygonaceae	Native	25
*Cirsium andersonii*	Asteraceae	Native	11
*Cirsium vulgare*	Asteraceae	Invasive	198
*Cuscuta californica*	Convolvulaceae	Native	50
*Drymacallis glandulosa*	Rosaceae	Native	13
*Eriodictyon lobbii*	Boraginaceae	Native	50
*Eriophyllum lanatum*	Asteraceae	Native	17
*Helenium bigelovii*	Asteraceae	Native	21
*Horkelia fusca*	Rosaceae	Native	51
*Hypericum perforatum*	Hypericaceae	Invasive	19
*Hypericum scouleri*	Hypericaceae	Native	11
*Lupinus latifolius var. columbianus*	Fabaceae	Native	13
*Lupinus* species	Fabaceae	Native	70
*Mimulus guttatus*	Phrymaceae	Native	17
*Mimulus moschatus*	Phrymaceae	Native	16
*Monardella odoratissima*	Lamiaceae	Native	14
*Oreostemma alpigenum var. andersonii*	Asteraceae	Native	15
*Perideridia parishii*	Apiaceae	Native	11
*Phacelia hastata*	Boraginaceae	Native	13
*Phacelia* species	Boraginaceae	Native	30
*Rudbeckia occidentalis*	Asteraceae	Native	10
*Senecio triangularis*	Asteraceae	Native	23
*Sidalcea glaucescens*	Malvaceae	Native	18
*Solidago canadensis*	Asteraceae	Native	19
*Solidago elongata*	Asteraceae	Native	13
*Symphyotrichum spathulatum*	Asteraceae	Native	122
*Trifolium pratense*	Fabaceae	Non‐Native	44
*Veratrum californicum*	Melanthiaceae	Native	21

**TABLE A4:** Tests for correlations among predictor variables used to model pollinator abundance with Pearson's correlation and variance inflation factor (VIF), stratified by upland and meadow habitat.

Pearson correlations	Burn severity	Floral abundance	Variables	VIF
Upland				
Floral abundance	0.013		Burn severity	1.019
Floral richness	0.128	0.454	Floral abundance	1.264
			Floral richness	1.284
Meadow				
Floral abundance	0.203		Burn severity	1.092
Floral richness	0.290	0.676	Floral abundance	1.842
			Floral richness	1.928
All				
Floral abundance	0.024		Burn severity	1.293
Floral richness	0.163	0.397	Floral abundance	1.190
			Floral richness	1.221

*Note*: Data were collected in the Sierra Nevada, CA, 2 and 3 years after the 2014 King Fire. Floral abundance and richness were derived from the 30 most commonly visited plant species (Table A1). Elevation and burn severity were derived from remote‐sensed data.

**TABLE A5:** Abundance of pollinator species or morphospecies detected in the Sierra Nevada of California 2 and 3 years after the 2014 King Fire.

Order	Family	Scientific name	Code	GU	MU	HU	GM	HM
Apodiformes	Trochilidae	NA	NA	1	4	11	9	30
Coleoptera	Cerambycidae	*Anastrangalia sanguinea*	A.sang	0	1	0	2	4
Coleoptera	Cerambycidae	Cerambycidae	CERAM	0	0	0	1	2
Coleoptera	Cerambycidae	*Lepturobosca chrysocoma*	L.chry	0	0	0	3	0
Coleoptera	Dermestidae	*Orphilus subnitidus*	O.subn	0	2	0	1	1
Coleoptera	Mordellidae	Mordella 1	MORDEL	0	4	0	1	1
Diptera	Bombyliidae	Bombyliidae 4	BOMBY4	1	2	0	1	0
Diptera	Bombyliidae	Bombyliidae 6	BOMBY6	0	0	1	0	1
Diptera	Bombyliidae	Geron	GERON	0	1	0	0	0
Diptera	Syrphidae	Syrphidae 3	SYRPH3	0	2	1	5	1
Diptera	Syrphidae	Syrphidae 4	SYRPH4	0	0	1	5	0
Diptera	Tachinidae	Tachinidae 1	TACHI1	0	0	0	1	2
Diptera	Tachinidae	Tachinidae 3	TACHI3	0	0	0	2	2
Hemiptera	Miridae	*Lygus* spp.	LYGUS	0	1	3	1	1
Hymenoptera	Andrenidae	*Calliopsis edwardsii*	C.edwa	0	1	0	7	1
Hymenoptera	Apidae	*Anthophora urbana*	A.urba	4	11	0	7	6
Hymenoptera	Apidae	*Apis mellifera*	A.mell	4	39	25	24	11
Hymenoptera	Apidae	*Bombus bifarius*	B.bifa	0	0	0	19	0
Hymenoptera	Apidae	*Bombus fernaldae*	B.fern	1	0	0	0	4
Hymenoptera	Apidae	*Bombus fervidus*	B.ferv	2	0	2	3	29
Hymenoptera	Apidae	*Bombus flavifrons*	B.flav	4	1	2	8	9
Hymenoptera	Apidae	*Bombus insularis*	B.insu	1	1	1	4	8
Hymenoptera	Apidae	*Bombus melanopygus*	B.mela	2	0	0	1	2
Hymenoptera	Apidae	*Bombus mixtus*	B.mixt	6	1	0	22	1
Hymenoptera	Apidae	*Bombus rufocinctus*	B.rufo	0	0	0	3	3
Hymenoptera	Apidae	*Bombus vandykei*	B.vand	1	6	16	4	37
Hymenoptera	Apidae	*Bombus vosnesenskii*	B.vosn	20	27	63	208	513
Hymenoptera	Apidae	*Melissodes microsticta*	M.micr	0	0	0	2	1
Hymenoptera	Apidae	*Xeromelecta californica*	X.cali	0	0	0	1	3
Hymenoptera	Collectidae	*Hylaeus episcopalis*	H.epis	1	0	0	0	2
Hymenoptera	Crabronidae	Crabronidae 1	CRABO1	0	0	2	0	1
Hymenoptera	Halictidae	Halictidae	HALIC	0	2	1	1	0
Hymenoptera	Halictidae	*Halictus confusus*	H.conf	3	2	0	1	5
Hymenoptera	Halictidae	Lasioglossum 2	LASIG2	0	1	0	1	1
Hymenoptera	Halictidae	*Lasioglossum anhypops*	L.anhy	0	2	0	1	0
Hymenoptera	Halictidae	*Lasioglossum dialictus*	L.Dial	1	4	11	8	3
Hymenoptera	Halictidae	*Lasioglossum olympiae*	L.olym	0	0	0	8	4
Hymenoptera	Megachilidae	*Megachile angelarum*	M.ange	0	2	2	1	0
Hymenoptera	Megachilidae	*Osmia coloradensis*	O.colo	0	1	1	0	1
Hymenoptera	Megachilidae	*Osmia montana*	O.mont	0	0	1	0	2
Hymenoptera	Sphecidae	*Podalonia*	PODALO	0	0	2	0	2
Lepidoptera	Hesperiidae	NA	NA	3	NA	3	16	6
Lepidoptera	Lycaenidae	NA	NA	2	NA	22	4	21
Lepidoptera	Nymphalidae	NA	NA	6	NA	59	23	61
Lepidoptera	Papilionidae	NA	NA	0	NA	0	0	2
Lepidoptera	Pieridae	NA	NA	1	NA	63	21	108

*Note*: Moderate‐severity habitat was only sampled in 2017. Butterflies were only sampled in 2016, and other insects (bees, wasps, flies, true bugs, and beetles) were only sampled in 2017. Different taxa were sampled with different methodologies (see main text for details).

Abbreviations: Code, species code used in figures; GU, unburned upland; HU, high‐severity upland; HM, high‐severity meadow; MU, moderate‐severity upland.

## APPENDIX B

### B.1

We used Bayesian *p*‐values to summarize the posterior predictive check for the goodness‐of‐fit of our models. We defined our test statistic chi‐square as:

$$\chi^{2}=\frac{Y_{j}-\left(\lambda_{j}*\rho \right)2}{\sqrt{\lambda_{j}*\rho }+e}$$

where $Y_{j}$ are the counts by site j, $\lambda_{j}$ is our mean abundance, ρ is the overdispersion parameter, and e = 0.0001. This was summed over all observations to generate the posterior distribution of our observed dataset.

We created a hypothetical perfect dataset that followed the Poisson distribution with parameter $\lambda_{j}*\rho$ statistic, with the posterior distribution and calculated the posterior distribution of this “expected” dataset using the equation above. Both are expected and observed chi‐square statistics are calculated in each run of the MCMC with the respective parameter estimates. The Bayesian *p*‐value is the posterior probability of observing a more extreme value, given the data (Gelman et al., 1996; Kéry & Royle, 2020).

**TABLE B1:** Posterior means and standard deviations (SD) of coefficients related to habitat, fire, and floral resources from negative binomial generalized linear mixed models (community models for *Bombus* and insects, regular models for butterflies and hummingbirds).

Taxon	Code	Burn severity in meadow		Burn severity in upland		Burn severity^2^ in upland		Habitat, upland		Floral abundance		Floral richness	
		Mean	SD	Mean	SD	Mean	SD	Mean	SD	Mean	SD	Mean	SD
*Bombus* community		−0.525	0.49	−0.206	0.603	−0.695	0.569	**−2.121**	**0.701**	−0.023	0.19	**0.468**	**0.199**
*Bombus bifarius*	BOBI	−1.340	0.969	−0.897	1.150	−1.070	1.093	**−2.545**	**1.316**	−0.086	0.285	0.280	0.328
*Bombus fervidus*	BOFE	−0.287	0.508	0.070	0.605	−0.519	0.704	**−2.383**	**1.007**	0.127	0.214	**0.570**	**0.254**
*Bombus flavifrons*	BOFL	−0.161	0.508	0.452	0.634	−0.089	0.780	**−2.404**	**1.072**	0.052	0.228	0.243	0.286
*Bombus insularis*	BOIN	−0.401	0.496	−0.222	0.671	**−1.402**	**0.948**	**−2.132**	**0.844**	−0.176	0.303	0.353	0.279
*Bombus mixtus*	BOMI	**−1.547**	**0.814**	−1.686	0.972	−0.550	0.768	**−1.948**	**0.957**	−0.110	0.288	**0.595**	**0.271**
*Bombus vandykei*	BOVA	0.362	0.463	0.582	0.433	−0.780	0.511	−1.466	0.812	−0.063	0.193	**0.562**	**0.187**
*Bombus vosnesenskii*	BOVO	−0.161	0.335	0.444	0.261	−0.581	0.362	**−1.919**	**0.578**	0.127	0.156	**0.646**	**0.143**
Insect community		**−0.551**	**0.259**	0.199	0.266	**−0.628**	**0.319**	**−2.028**	**0.474**	0.000	0.105	**0.389**	**0.117**
*Anastrangalia sanguinea*	ANSA	−0.745	0.480	−0.191	0.693	−0.776	0.593	**−2.331**	**0.915**	−0.060	0.251	0.268	0.272
*Anthophora urbana*	ANUR	−0.590	0.410	−0.308	0.495	−0.846	0.519	−1.343	0.796	0.094	0.201	0.366	0.22
*Apis mellifera*	APME	−0.389	0.382	0.541	0.34	−0.528	0.375	−1.159	0.703	0.141	0.114	**0.664**	**0.189**
*Bombyliidae 4*	Bomb4	−0.680	0.456	−0.183	0.624	−0.439	0.554	−1.591	0.839	0.048	0.233	0.441	0.249
*Bombyliidae 6*	Bomb6	−0.283	0.467	0.777	0.72	−0.499	0.558	**−2.062**	**0.890**	−0.018	0.252	0.37	0.268
*Calliopsis edwardsii*	CAED	−0.742	0.482	−0.103	0.698	−0.780	0.594	**−2.435**	**0.967**	0.018	0.226	0.322	0.269
*Cerambycidae*	CERAM	−0.616	0.457	0.135	0.722	−0.790	0.612	**−2.539**	**1.042**	0.042	0.228	0.321	0.27
*Crabronidae 1*	CRAB1	−0.329	0.451	0.929	0.722	−0.349	0.562	**−1.842**	**0.862**	−0.076	0.262	0.357	0.271
*Geron*	GERON	−0.661	0.474	−0.219	0.759	−0.569	0.577	**−1.984**	**0.933**	−0.041	0.259	0.330	0.283
*Halictus confusus*	HACO	−0.410	0.403	−0.077	0.59	−0.766	0.526	**−1.724**	**0.767**	−0.065	0.237	**0.493**	**0.24**
*Halictidae*	HALI	−0.570	0.444	0.164	0.627	−0.312	0.567	−1.662	0.861	0.253	0.161	0.306	0.27
*Hylaeus episcopalis*	HYEP	−0.495	0.448	0.138	0.716	−0.816	0.607	**−2.188**	**0.969**	−0.064	0.266	0.330	0.286
*Lasioglossum anhypops*	LAAN	−0.769	0.491	−0.552	0.747	−0.428	0.563	**−1.904**	**0.872**	−0.117	0.261	0.284	0.27
*Lasioglossum Dialictus*	LADI	−0.516	0.388	0.664	0.454	−0.692	0.45	**−1.362**	**0.677**	0.02	0.201	**0.501**	**0.203**
*Lasioglossum olympiae*	LAOL	−0.663	0.451	0.126	0.688	−0.876	0.628	**−2.822**	**1.094**	0.019	0.213	0.277	0.252
*Lasioglossum 2*	LASI2	−0.650	0.466	−0.196	0.722	−0.601	0.566	**−2.15**	**0.891**	0.002	0.230	0.323	0.269
*Lepturobosca chrysocoma*	LECH	−0.607	0.462	0.15	0.724	−0.787	0.614	**−2.528**	**1.05**	−0.107	0.264	0.313	0.262
*Lygus* spp.	LYGUS	−0.509	0.439	0.565	0.646	−0.492	0.542	**−1.941**	**0.899**	−0.013	0.234	0.288	0.263
*Megachile angelarum*	MEAN	−0.435	0.432	0.717	0.609	−0.479	0.522	−1.355	0.866	−0.042	0.242	0.482	0.248
*Melissodes microsticta*	MEMI	−0.494	0.438	0.245	0.714	−0.794	0.612	**−2.525**	**1.050**	−0.012	0.247	0.416	0.269
*Mordella 1*	MORD1	−0.694	0.465	−0.541	0.729	−0.528	0.55	**−1.951**	**0.917**	−0.049	0.233	**0.561**	**0.254**
*Orphilus subnitidus*	ORSU	−0.613	0.441	0.216	0.614	−0.520	0.543	**−1.933**	**0.844**	−0.009	0.233	0.415	0.258
*Osmia coloradensis*	OSCO	−0.441	0.445	0.515	0.65	−0.485	0.542	**−1.766**	**0.847**	0.017	0.232	0.363	0.267
*Osmia montana*	OSMO	−0.609	0.458	0.086	0.698	−0.850	0.613	**−2.146**	**0.902**	0.078	0.227	0.430	0.267
*Podalonia*	PODAL	−0.247	0.457	0.989	0.718	−0.304	0.559	**−1.89**	**0.847**	−0.053	0.258	0.429	0.266
*Syrphidae 3*	SYR3	−0.618	0.426	0.221	0.594	−0.667	0.532	**−1.799**	**0.764**	−0.092	0.242	**0.540**	**0.237**
*Syrphidae 4*	SYR4	−0.526	0.441	0.601	0.68	−0.498	0.552	**−2.251**	**0.881**	0.066	0.231	0.329	0.262
*Tachinidae 1*	TACH1	−0.626	0.457	0.128	0.723	−0.795	0.611	**−2.541**	**1.052**	0.059	0.231	0.333	0.273
*Tachinidae 3*	TACH3	−0.591	0.457	0.144	0.726	−0.804	0.615	**−2.567**	**1.055**	0.072	0.221	0.416	0.254
*Xeromelecta californica*	XECA	−0.379	0.437	0.331	0.722	−0.792	0.607	**−2.492**	**1.035**	−0.100	0.265	0.434	0.271
Butterfly													
Hesperiidae		**−0.464**	**0.719**	0.435	0.852	NA	NA	**−3.261**	**1.296**	0.677	0.352	0.089	0.400
Lycaenidae		0.799	0.739	**2.082**	**0.829**	NA	NA	**−2.297**	**1.124**	0.712	0.517	0.369	0.381
Nymphalidae		0.159	0.420	0.576	0.418	NA	NA	−1.380	0.753	0.146	0.185	0.247	0.193
Pieridae		0.776	0.456	**1.872**	**0.534**	NA	NA	**−2.710**	**0.839**	0.146	0.193	−0.190	0.194
Hummingbird		**0.917**	**0.410**	0.833	0.445	−0.225	0.684	**−1.899**	**0.971**	**0.218**	**0.107**	**0.375**	**0.160**

*Note*: Data were collected in upland and meadow habitat in the Sierra Nevada, CA, in 2016 and 2017, following the 2014 King Fire. Full model results are available in Tables B2, B3, B4, B5. Bold values have 95% Bayesian credible intervals that do not overlap zero and are considered significant. Code = species code used in figures.

**TABLE B2:** Posterior means, standard deviations (SD), and lower (2.50%) and upper (97.50%) limits of 95% Bayesian credible interval and convergence statistic (Rhat; <1.1 indicates convergence) of parameters from the Bumblebee community negative binomial generalized linear mixed model.

Parameter	Response	Covariate	Mean	SD	2.50%	97.50%	Rhat
Beta	Community	Intercept	−3.016	1.093	−5.446	−1.090	1.062
Beta	Community	DSS	−0.075	0.224	−0.522	0.375	1.003
Beta	Community	Floral richness	0.468	0.199	0.060	0.852	1.007
Beta	Community	Floral abundance	−0.023	0.190	−0.439	0.319	1.006
Beta	Community	Year	−0.354	0.870	−2.124	1.392	1.012
Beta	Community	Elevation (m)	0.208	0.374	−0.490	0.990	1.021
beta	Community	Burn severity, meadow	−0.525	0.490	−1.676	0.323	1.005
Beta	Community	Habitat, upland	−2.121	0.701	−3.693	−0.885	1.001
Beta	Community	Burn (Upland‐Meadow)	0.319	0.489	−0.655	1.228	1.004
Beta	Community	Burn severity^2^, upland	−0.695	0.569	−1.864	0.347	1.011
Delta	BOBI	Intercept	−2.106	1.726	−6.172	0.748	1.011
Delta	BOFE	Intercept	−1.324	1.489	−4.618	1.492	1.014
Delta	BOFL	Intercept	−1.176	1.480	−4.361	1.512	1.016
Delta	BOMI	Intercept	−0.336	1.333	−3.039	2.271	1.021
Delta	BOVA	Intercept	1.355	1.200	−0.918	3.938	1.039
Delta	BOVO	Intercept	4.239	1.125	2.295	6.788	1.063
Delta	BOIN	Intercept	−2.121	1.509	−5.345	0.647	1.013
Delta	BOBI	DSS	0.022	0.346	−0.672	0.724	1.000
Delta	BOFE	DSS	−0.069	0.311	−0.714	0.536	1.000
Delta	BOFL	DSS	0.465	0.343	−0.119	1.229	1.001
Delta	BOMI	DSS	−0.302	0.336	−1.040	0.293	1.000
Delta	BOVA	DSS	−0.084	0.277	−0.648	0.452	1.001
Delta	BOVO	DSS	−0.051	0.247	−0.557	0.434	1.003
Delta	BOIN	DSS	0.028	0.306	−0.575	0.649	1.001
Delta	BOBI	Floral richness	−0.187	0.314	−0.875	0.383	1.000
Delta	BOFE	Floral richness	0.102	0.273	−0.419	0.678	1.001
Delta	BOFL	Floral richness	−0.225	0.287	−0.845	0.292	1.001
Delta	BOMI	Floral richness	0.127	0.284	−0.400	0.735	1.001
Delta	BOVA	Floral richness	0.094	0.238	−0.369	0.585	1.004
Delta	BOVO	Floral richness	0.178	0.216	−0.223	0.630	1.005
Delta	BOIN	Floral richness	−0.115	0.281	−0.705	0.420	1.001
Delta	BOBI	Floral abundance	−0.063	0.279	−0.639	0.477	1.001
Delta	BOFE	Floral abundance	0.150	0.247	−0.305	0.685	1.003
Delta	BOFL	Floral abundance	0.075	0.250	−0.405	0.595	1.002
Delta	BOMI	Floral abundance	−0.087	0.277	−0.680	0.429	1.000
Delta	BOVA	Floral abundance	−0.040	0.227	−0.486	0.420	1.002
Delta	BOVO	Floral abundance	0.150	0.221	−0.247	0.640	1.005
Delta	BOIN	Floral abundance	−0.153	0.288	−0.792	0.361	1.000
Delta	BOBI	Year	−1.627	1.183	−4.140	0.540	1.004
Delta	BOFE	Year	1.140	1.005	−0.797	3.225	1.008
Delta	BOFL	Year	0.552	1.006	−1.429	2.622	1.005
Delta	BOMI	Year	−1.728	1.254	−4.463	0.474	1.006
Delta	BOVA	Year	−0.451	0.961	−2.383	1.472	1.008
Delta	BOVO	Year	−1.199	0.912	−3.060	0.624	1.012
Delta	BOIN	Year	3.292	1.317	1.056	6.265	1.004
Delta	BOBI	Elevation (m)	0.782	0.718	−0.348	2.371	1.002
Delta	BOFE	Elevation (m)	−0.487	0.595	−1.832	0.489	1.005
Delta	BOFL	Elevation (m)	−0.002	0.505	−1.061	1.011	1.003
Delta	BOMI	Elevation (m)	0.030	0.499	−0.929	1.097	1.006
Delta	BOVA	Elevation (m)	0.148	0.449	−0.728	1.086	1.007
Delta	BOVO	Elevation (m)	−0.073	0.408	−0.941	0.700	1.019
Delta	BOIN	Elevation (m)	−0.367	0.500	−1.465	0.525	1.011
Delta	BOBI	Burn severity, meadow	−0.815	0.902	−2.954	0.525	1.005
Delta	BOFE	Burn severity, meadow	0.238	0.607	−0.855	1.593	1.003
Delta	BOFL	Burn severity, meadow	0.364	0.622	−0.733	1.769	1.002
Delta	BOMI	Burn severity, meadow	−1.022	0.773	−2.757	0.227	1.001
Delta	BOVA	Burn severity, meadow	0.887	0.627	−0.147	2.323	1.003
Delta	BOVO	Burn severity, meadow	0.364	0.535	−0.550	1.616	1.017
Delta	BOIN	Burn severity, meadow	0.124	0.594	−0.995	1.418	1.002
Delta	BOBI	Habitat, upland	−0.424	1.067	−3.127	1.130	1.002
Delta	BOFE	Habitat, upland	−0.261	0.831	−2.256	1.239	1.001
Delta	BOFL	Habitat, upland	−0.283	0.866	−2.375	1.190	1.003
Delta	BOMI	Habitat, upland	0.173	0.830	−1.450	2.067	1.001
Delta	BOVA	Habitat, upland	0.656	0.871	−0.597	2.842	1.002
Delta	BOVO	Habitat, upland	0.202	0.684	−0.977	1.943	1.003
Delta	BOIN	Habitat, upland	−0.011	0.766	−1.570	1.647	1.001
Delta	BOBI	Burn (Upland‐Meadow)	0.125	0.705	−1.182	1.712	1.001
Delta	BOFE	Burn (Upland‐Meadow)	0.039	0.570	−1.076	1.272	1.001
Delta	BOFL	Burn (Upland‐Meadow)	0.294	0.636	−0.749	1.806	1.002
Delta	BOMI	Burn (Upland‐Meadow)	−0.458	0.750	−2.261	0.634	1.001
Delta	BOVA	Burn (Upland‐Meadow)	−0.099	0.537	−1.196	0.960	1.002
Delta	BOVO	Burn (Upland‐Meadow)	0.286	0.500	−0.588	1.376	1.011
Delta	BOIN	Burn (Upland‐Meadow)	−0.140	0.602	−1.465	0.988	1.001
Delta	BOBI	Burn severity^2^, upland	−0.375	0.996	−2.844	1.082	1.009
Delta	BOFE	Burn severity^2^, upland	0.176	0.692	−1.131	1.731	1.003
Delta	BOFL	Burn severity^2^, upland	0.606	0.789	−0.559	2.591	1.001
Delta	BOMI	Burn severity^2^, upland	0.145	0.734	−1.314	1.764	1.003
Delta	BOVA	Burn severity^2^, upland	−0.085	0.616	−1.381	1.155	1.012
Delta	BOVO	Burn severity^2^, upland	0.114	0.572	−0.926	1.351	1.013
Delta	BOIN	Burn severity^2^, upland	−0.707	0.905	−3.037	0.487	1.009
Sigma	Community	Intercept	2.776	1.087	1.387	5.497	1.003
Sigma	Community	DSS	0.436	0.203	0.187	0.945	1.001
Sigma	Community	Floral richness	0.377	0.169	0.172	0.803	1.002
Sigma	Community	Floral abundance	0.347	0.155	0.163	0.739	1.002
Sigma	Community	Year	2.157	0.912	0.966	4.438	1.006
Sigma	Community	Elevation (m)	0.680	0.389	0.215	1.661	1.005
Sigma	Community	Burn severity, meadow	0.954	0.533	0.280	2.297	1.002
Sigma	Community	Habitat, upland	0.863	0.650	0.220	2.575	1.003
Sigma	Community	Burn (Upland‐Meadow)	0.663	0.446	0.208	1.804	1.003
Sigma	Community	Burn severity^2^, upland	0.818	0.613	0.218	2.428	1.015
Sigma	BOBI	Model	1.701	1.363	0.067	5.160	1.002
Sigma	BOFE	Model	2.354	1.083	0.737	4.922	1.001
Sigma	BOFL	Model	2.587	0.984	1.119	4.945	1.004
Sigma	BOMI	Model	1.998	1.068	0.349	4.573	1.019
Sigma	BOVA	Model	1.434	0.541	0.546	2.673	1.001
Sigma	BOVO	Model	1.063	0.268	0.613	1.662	1.000
Sigma	BOIN	Model	1.091	0.787	0.043	2.966	1.003
BP, species	BOBI	Model	0.599	0.490	0.000	1.000	1.000
BP, species	BOFE	Model	0.379	0.485	0.000	1.000	1.000
BP, species	BOFL	Model	0.363	0.481	0.000	1.000	1.000
BP, species	BOMI	Model	0.369	0.482	0.000	1.000	1.000
BP, species	BOVA	Model	0.310	0.462	0.000	1.000	1.000
BP, species	BOVO	Model	0.500	0.500	0.000	1.000	1.000
BP, species	BOIN	Model	0.516	0.500	0.000	1.000	1.000
BP, community	Community	Model	0.353	0.478	0.000	1.000	1.000
*r*	Community	Model	0.312	0.041	0.240	0.401	1.000

*Note*: Data were collected in 2016 and 2017 in the Sierra Nevada, California, following the 2014 King Fire. Beta shows the community‐level response to the covariate, and delta indicates the deviation of the species from the community response. The values reported in the text for species‐level response were derived from actual MCMC chains. See Table A3 for species code interpretation.

Abbreviations: BP, Bayesian *p*‐value; DSS, days since snowmelt; *r*, negative binomial dispersion parameter; Sigma, standard deviation of random effect of site.

**TABLE B3:** Posterior means, standard deviations (SD), and lower (2.50%) and upper (97.50%) limits of 95% Bayesian credible interval and convergence statistic (Rhat; <1.1 indicates convergence) of parameters from the insect community negative binomial generalized linear mixed model.

Parameter	Response variable	Covariate	Mean	SD	2.50%	97.50%	Rhat
Beta	Community	Intercept	−4.061	0.353	−4.787	−3.416	1.002
Beta	Community	DSS	−0.585	0.180	−0.948	−0.236	1.001
Beta	Community	Floral richness	0.000	0.105	−0.223	0.192	1.000
Beta	Community	Floral abundance	0.389	0.117	0.159	0.616	1.000
Beta	Community	Elevation (m)	0.071	0.135	−0.197	0.333	1.000
Beta	Community	Burn severity, meadow	−0.551	0.259	−1.075	−0.056	1.000
Beta	Community	Habitat, upland	−2.028	0.474	−3.055	−1.186	1.002
Beta	Community	Burn (Upland‐Meadow)	0.749	0.344	0.081	1.432	1.001
Beta	Community	Burn severity^2^, upland	−0.628	0.319	−1.261	−0.002	1.000
Delta	ANSA	Intercept	−0.382	0.884	−2.322	1.183	1.004
Delta	CERAM	Intercept	−0.525	0.828	−2.281	0.979	1.000
Delta	LAAN	Intercept	−0.575	0.852	−2.418	0.949	1.000
Delta	LAOL	Intercept	0.208	0.829	−1.608	1.695	1.001
Delta	LECH	Intercept	−0.495	0.843	−2.324	1.016	1.001
Delta	SYR3	Intercept	0.750	0.757	−0.846	2.136	1.002
Delta	TACH3	Intercept	−0.547	0.853	−2.397	0.984	1.000
Delta	ANUR	Intercept	1.504	0.828	−0.161	3.035	1.001
Delta	APME	Intercept	2.740	0.862	0.803	4.283	1.005
Delta	XECA	Intercept	0.091	0.788	−1.573	1.542	1.000
Delta	LYGUS	Intercept	−0.223	0.883	−2.114	1.370	1.003
Delta	ORSU	Intercept	−0.212	0.792	−1.897	1.247	1.003
Delta	SYR4	Intercept	0.148	0.826	−1.673	1.623	1.003
Delta	Bomb4	Intercept	−0.333	0.851	−2.188	1.184	1.000
Delta	MEAN	Intercept	0.091	0.770	−1.530	1.529	1.002
Delta	LADI	Intercept	1.989	0.711	0.422	3.276	1.002
Delta	MEMI	Intercept	−0.034	0.794	−1.742	1.414	1.000
Delta	HACO	Intercept	1.126	0.796	−0.534	2.581	1.002
Delta	MORD1	Intercept	−0.348	0.880	−2.251	1.239	1.000
Delta	LASI2	Intercept	−0.436	0.822	−2.183	1.068	1.001
Delta	TACH1	Intercept	−0.143	0.803	−1.858	1.334	1.001
Delta	CAED	Intercept	−0.209	0.930	−2.236	1.459	1.000
Delta	HALI	Intercept	−0.403	0.857	−2.290	1.124	1.001
Delta	HYEP	Intercept	−0.656	0.976	−2.815	1.048	1.000
Delta	CRAB1	Intercept	−0.364	0.852	−2.228	1.154	1.000
Delta	OSMO	Intercept	−0.614	0.844	−2.425	0.922	1.000
Delta	Bomb6	Intercept	−0.604	0.887	−2.552	0.960	1.001
Delta	PODAL	Intercept	−0.168	0.783	−1.835	1.263	1.000
Delta	OSCO	Intercept	−0.517	0.856	−2.366	1.025	1.001
Delta	GERON	Intercept	−0.975	0.945	−3.053	0.649	1.000
Delta	ANSA	DSS	−0.939	0.516	−2.049	−0.025	1.000
Delta	CERAM	DSS	−0.684	0.522	−1.801	0.251	1.000
Delta	LAAN	DSS	−0.536	0.506	−1.603	0.399	1.000
Delta	LAOL	DSS	−0.880	0.467	−1.869	−0.043	1.000
Delta	LECH	DSS	−0.565	0.513	−1.648	0.367	1.000
Delta	SYR3	DSS	0.341	0.377	−0.384	1.102	1.000
Delta	TACH3	DSS	−0.663	0.520	−1.766	0.284	1.000
Delta	ANUR	DSS	0.818	0.336	0.183	1.498	1.001
Delta	APME	DSS	0.504	0.278	−0.032	1.052	1.001
Delta	XECA	DSS	0.916	0.519	−0.022	2.007	1.000
Delta	LYGUS	DSS	−0.307	0.465	−1.264	0.569	1.000
Delta	ORSU	DSS	−0.462	0.495	−1.495	0.453	1.000
Delta	SYR4	DSS	−0.066	0.444	−0.956	0.789	1.000
Delta	Bomb4	DSS	0.130	0.472	−0.800	1.067	1.000
Delta	MEAN	DSS	0.339	0.451	−0.523	1.255	1.000
Delta	LADI	DSS	0.086	0.314	−0.532	0.702	1.000
Delta	MEMI	DSS	0.164	0.490	−0.794	1.144	1.000
Delta	HACO	DSS	0.219	0.382	−0.530	0.980	1.000
Delta	MORD1	DSS	−0.066	0.469	−1.009	0.839	1.000
Delta	LASI2	DSS	−0.432	0.517	−1.517	0.520	1.000
Delta	TACH1	DSS	0.052	0.492	−0.929	1.016	1.000
Delta	CAED	DSS	−0.087	0.415	−0.917	0.717	1.000
Delta	HALI	DSS	0.473	0.494	−0.459	1.484	1.000
Delta	HYEP	DSS	0.769	0.570	−0.264	1.980	1.000
Delta	CRAB1	DSS	0.545	0.505	−0.396	1.597	1.000
Delta	OSMO	DSS	−0.602	0.542	−1.746	0.384	1.000
Delta	Bomb6	DSS	0.021	0.527	−1.027	1.062	1.000
Delta	PODAL	DSS	1.044	0.535	0.084	2.179	1.000
Delta	OSCO	DSS	−0.345	0.514	−1.408	0.615	1.000
Delta	GERON	DSS	0.142	0.556	−0.942	1.260	1.000
Delta	ANSA	Floral abundance	−0.060	0.228	−0.539	0.370	1.000
Delta	CERAM	Floral abundance	0.042	0.215	−0.381	0.475	1.000
Delta	LAAN	Floral abundance	−0.117	0.235	−0.630	0.308	1.000
Delta	LAOL	Floral abundance	0.019	0.201	−0.375	0.426	1.000
Delta	LECH	Floral abundance	−0.108	0.237	−0.620	0.326	1.000
Delta	SYR3	Floral abundance	−0.092	0.221	−0.562	0.318	1.000
Delta	TACH3	Floral abundance	0.072	0.212	−0.337	0.504	1.000
Delta	ANUR	Floral abundance	0.094	0.199	−0.287	0.503	1.000
Delta	APME	Floral abundance	0.141	0.142	−0.112	0.449	1.000
Delta	XECA	Floral abundance	−0.100	0.239	−0.615	0.335	1.000
Delta	LYGUS	Floral abundance	−0.013	0.217	−0.457	0.408	1.000
Delta	ORSU	Floral abundance	−0.009	0.218	−0.454	0.417	1.000
Delta	SYR4	Floral abundance	0.066	0.221	−0.368	0.514	1.000
Delta	Bomb4	Floral abundance	0.047	0.221	−0.390	0.490	1.000
Delta	MEAN	Floral abundance	−0.042	0.223	−0.510	0.378	1.000
Delta	LADI	Floral abundance	0.020	0.192	−0.359	0.403	1.000
Delta	MEMI	Floral abundance	−0.012	0.229	−0.480	0.433	1.000
Delta	HACO	Floral abundance	−0.065	0.217	−0.521	0.343	1.000
Delta	MORD1	Floral abundance	−0.049	0.214	−0.498	0.359	1.000
Delta	LASI2	Floral abundance	0.002	0.215	−0.433	0.420	1.000
Delta	TACH1	Floral abundance	0.059	0.219	−0.370	0.499	1.000
Delta	CAED	Floral abundance	0.018	0.213	−0.410	0.438	1.000
Delta	HALI	Floral abundance	0.253	0.179	−0.072	0.630	1.000
Delta	HYEP	Floral abundance	−0.064	0.242	−0.574	0.394	1.000
Delta	CRAB1	Floral abundance	−0.076	0.238	−0.582	0.367	1.000
Delta	OSMO	Floral abundance	0.078	0.218	−0.345	0.523	1.000
Delta	Bomb6	Floral abundance	−0.019	0.232	−0.493	0.430	1.000
Delta	PODAL	Floral abundance	−0.053	0.235	−0.548	0.390	1.000
Delta	OSCO	Floral abundance	0.017	0.217	−0.420	0.449	1.000
Delta	GERON	Floral abundance	−0.041	0.237	−0.535	0.408	1.000
Delta	ANSA	Floral richness	−0.121	0.251	−0.654	0.348	1.000
Delta	CERAM	Floral richness	−0.068	0.249	−0.584	0.408	1.000
Delta	LAAN	Floral richness	−0.106	0.248	−0.633	0.358	1.000
Delta	LAOL	Floral richness	−0.112	0.233	−0.594	0.332	1.000
Delta	LECH	Floral richness	−0.076	0.242	−0.575	0.391	1.000
Delta	SYR3	Floral richness	0.150	0.228	−0.272	0.629	1.000
Delta	TACH3	Floral richness	0.027	0.238	−0.441	0.508	1.000
Delta	ANUR	Floral richness	−0.023	0.209	−0.437	0.393	1.000
Delta	APME	Floral richness	0.274	0.198	−0.084	0.691	1.000
Delta	XECA	Floral richness	0.045	0.253	−0.448	0.564	1.000
Delta	LYGUS	Floral richness	−0.101	0.243	−0.610	0.357	1.000
Delta	ORSU	Floral richness	0.026	0.242	−0.447	0.515	1.000
Delta	SYR4	Floral richness	−0.061	0.243	−0.561	0.407	1.000
Delta	Bomb4	Floral richness	0.052	0.234	−0.404	0.531	1.000
Delta	MEAN	Floral richness	0.093	0.235	−0.355	0.582	1.000
Delta	LADI	Floral richness	0.112	0.200	−0.265	0.527	1.000
Delta	MEMI	Floral richness	0.027	0.250	−0.465	0.534	1.000
Delta	HACO	Floral richness	0.104	0.230	−0.331	0.585	1.000
Delta	MORD1	Floral richness	0.172	0.244	−0.273	0.695	1.000
Delta	LASI2	Floral richness	−0.066	0.248	−0.580	0.413	1.000
Delta	TACH1	Floral richness	−0.056	0.252	−0.578	0.433	1.000
Delta	CAED	Floral richness	−0.068	0.248	−0.578	0.410	1.000
Delta	HALI	Floral richness	−0.083	0.249	−0.604	0.395	1.000
Delta	HYEP	Floral richness	−0.059	0.263	−0.604	0.450	1.000
Delta	CRAB1	Floral richness	−0.032	0.251	−0.543	0.463	1.000
Delta	OSMO	Floral richness	0.041	0.250	−0.448	0.552	1.000
Delta	Bomb6	Floral richness	−0.019	0.248	−0.519	0.474	1.000
Delta	PODAL	Floral richness	0.040	0.249	−0.448	0.548	1.000
Delta	OSCO	Floral richness	−0.026	0.247	−0.525	0.458	1.000
Delta	GERON	Floral richness	−0.059	0.261	−0.599	0.444	1.000
Delta	ANSA	Elevation (m)	−0.079	0.321	−0.742	0.546	1.000
Delta	CERAM	Elevation (m)	−0.139	0.339	−0.868	0.492	1.000
Delta	LAAN	Elevation (m)	0.024	0.319	−0.605	0.674	1.000
Delta	LAOL	Elevation (m)	0.283	0.309	−0.275	0.944	1.000
Delta	LECH	Elevation (m)	0.121	0.315	−0.476	0.782	1.000
Delta	SYR3	Elevation (m)	0.145	0.280	−0.385	0.730	1.000
Delta	TACH3	Elevation (m)	−0.033	0.316	−0.678	0.592	1.000
Delta	ANUR	Elevation (m)	0.310	0.273	−0.185	0.884	1.001
Delta	APME	Elevation (m)	−0.211	0.221	−0.658	0.215	1.000
Delta	XECA	Elevation (m)	−0.212	0.337	−0.949	0.399	1.000
Delta	LYGUS	Elevation (m)	−0.141	0.325	−0.830	0.472	1.000
Delta	ORSU	Elevation (m)	0.030	0.314	−0.588	0.668	1.000
Delta	SYR4	Elevation (m)	0.260	0.303	−0.290	0.904	1.000
Delta	Bomb4	Elevation (m)	0.000	0.316	−0.636	0.630	1.000
Delta	MEAN	Elevation (m)	−0.024	0.309	−0.650	0.589	1.000
Delta	LADI	Elevation (m)	0.005	0.240	−0.472	0.481	1.000
Delta	MEMI	Elevation (m)	−0.092	0.321	−0.763	0.525	1.000
Delta	HACO	Elevation (m)	−0.345	0.330	−1.056	0.246	1.001
Delta	MORD1	Elevation (m)	0.193	0.323	−0.395	0.899	1.000
Delta	LASI2	Elevation (m)	0.158	0.325	−0.450	0.848	1.000
Delta	TACH1	Elevation (m)	0.094	0.325	−0.529	0.778	1.000
Delta	CAED	Elevation (m)	0.354	0.356	−0.254	1.149	1.003
Delta	HALI	Elevation (m)	0.098	0.320	−0.513	0.769	1.000
Delta	HYEP	Elevation (m)	−0.012	0.341	−0.703	0.674	1.000
Delta	CRAB1	Elevation (m)	−0.204	0.350	−0.970	0.432	1.000
Delta	OSMO	Elevation (m)	−0.067	0.336	−0.766	0.587	1.000
Delta	Bomb6	Elevation (m)	−0.164	0.351	−0.920	0.482	1.000
Delta	PODAL	Elevation (m)	−0.204	0.344	−0.958	0.421	1.000
Delta	OSCO	Elevation (m)	−0.027	0.330	−0.695	0.631	1.000
Delta	GERON	Elevation (m)	−0.106	0.345	−0.837	0.552	1.000
Delta	ANSA	Burn severity, meadow	−0.194	0.401	−1.102	0.519	1.000
Delta	CERAM	Burn severity, meadow	−0.065	0.388	−0.879	0.697	1.000
Delta	LAAN	Burn severity, meadow	−0.218	0.410	−1.159	0.491	1.000
Delta	LAOL	Burn severity, meadow	−0.112	0.379	−0.939	0.602	1.000
Delta	LECH	Burn severity, meadow	−0.056	0.390	−0.885	0.702	1.000
Delta	SYR3	Burn severity, meadow	−0.067	0.358	−0.816	0.638	1.000
Delta	TACH3	Burn severity, meadow	−0.040	0.387	−0.850	0.723	1.000
Delta	ANUR	Burn severity, meadow	−0.039	0.343	−0.745	0.647	1.000
Delta	APME	Burn severity, meadow	0.162	0.328	−0.446	0.864	1.001
Delta	XECA	Burn severity, meadow	0.171	0.386	−0.526	1.030	1.000
Delta	LYGUS	Burn severity, meadow	0.042	0.373	−0.696	0.822	1.000
Delta	ORSU	Burn severity, meadow	−0.062	0.368	−0.842	0.655	1.000
Delta	SYR4	Burn severity, meadow	0.025	0.374	−0.713	0.804	1.000
Delta	Bomb4	Burn severity, meadow	−0.129	0.379	−0.953	0.578	1.000
Delta	MEAN	Burn severity, meadow	0.115	0.372	−0.583	0.924	1.000
Delta	LADI	Burn severity, meadow	0.035	0.332	−0.622	0.719	1.000
Delta	MEMI	Burn severity, meadow	0.057	0.377	−0.680	0.852	1.000
Delta	HACO	Burn severity, meadow	0.141	0.352	−0.516	0.900	1.000
Delta	MORD1	Burn severity, meadow	−0.144	0.389	−1.005	0.577	1.000
Delta	LASI2	Burn severity, meadow	−0.099	0.391	−0.952	0.640	1.000
Delta	TACH1	Burn severity, meadow	−0.075	0.385	−0.904	0.671	1.000
Delta	CAED	Burn severity, meadow	−0.191	0.405	−1.112	0.526	1.000
Delta	HALI	Burn severity, meadow	−0.019	0.375	−0.790	0.735	1.000
Delta	HYEP	Burn severity, meadow	0.056	0.382	−0.696	0.862	1.000
Delta	CRAB1	Burn severity, meadow	0.222	0.400	−0.474	1.131	1.000
Delta	OSMO	Burn severity, meadow	−0.058	0.386	−0.878	0.693	1.000
Delta	Bomb6	Burn severity, meadow	0.268	0.418	−0.436	1.244	1.000
Delta	PODAL	Burn severity, meadow	0.304	0.414	−0.388	1.269	1.000
Delta	OSCO	Burn severity, meadow	0.110	0.382	−0.611	0.940	1.000
Delta	GERON	Burn severity, meadow	−0.110	0.400	−0.984	0.635	1.000
Delta	ANSA	Habitat, upland	−0.303	0.782	−2.067	1.115	1.000
Delta	CERAM	Habitat, upland	−0.511	0.881	−2.618	0.934	1.000
Delta	LAAN	Habitat, upland	0.124	0.787	−1.473	1.792	1.000
Delta	LAOL	Habitat, upland	−0.794	0.922	−3.017	0.570	1.000
Delta	LECH	Habitat, upland	−0.500	0.885	−2.609	0.945	1.000
Delta	SYR3	Habitat, upland	0.229	0.718	−1.128	1.803	1.000
Delta	TACH3	Habitat, upland	−0.539	0.891	−2.670	0.888	1.001
Delta	ANUR	Habitat, upland	0.685	0.825	−0.679	2.564	1.001
Delta	APME	Habitat, upland	0.870	0.777	−0.365	2.626	1.001
Delta	XECA	Habitat, upland	−0.464	0.878	−2.561	0.990	1.000
Delta	LYGUS	Habitat, upland	0.087	0.806	−1.583	1.758	1.001
Delta	ORSU	Habitat, upland	0.095	0.768	−1.456	1.728	1.001
Delta	SYR4	Habitat, upland	−0.223	0.761	−1.907	1.219	1.000
Delta	Bomb4	Habitat, upland	0.438	0.818	−0.996	2.309	1.001
Delta	MEAN	Habitat, upland	0.673	0.869	−0.735	2.685	1.000
Delta	LADI	Habitat, upland	0.666	0.721	−0.508	2.295	1.001
Delta	MEMI	Habitat, upland	−0.497	0.892	−2.627	0.952	1.000
Delta	HACO	Habitat, upland	0.304	0.742	−1.068	1.952	1.001
Delta	MORD1	Habitat, upland	0.078	0.827	−1.621	1.801	1.000
Delta	LASI2	Habitat, upland	−0.121	0.775	−1.804	1.399	1.000
Delta	TACH1	Habitat, upland	−0.513	0.886	−2.629	0.938	1.000
Delta	CAED	Habitat, upland	−0.407	0.819	−2.302	1.012	1.000
Delta	HALI	Habitat, upland	0.366	0.818	−1.118	2.224	1.000
Delta	HYEP	Habitat, upland	−0.159	0.848	−2.038	1.481	1.000
Delta	CRAB1	Habitat, upland	0.187	0.794	−1.354	1.921	1.000
Delta	OSMO	Habitat, upland	−0.118	0.785	−1.833	1.409	1.000
Delta	Bomb6	Habitat, upland	−0.034	0.788	−1.700	1.567	1.000
Delta	PODAL	Habitat, upland	0.138	0.772	−1.383	1.787	1.000
Delta	OSCO	Habitat, upland	0.262	0.791	−1.233	2.009	1.000
Delta	GERON	Habitat, upland	0.044	0.834	−1.687	1.780	1.000
Delta	ANSA	Burn (Upland‐Meadow)	−0.196	0.592	−1.525	0.901	1.000
Delta	CERAM	Burn (Upland‐Meadow)	0.001	0.603	−1.232	1.256	1.000
Delta	LAAN	Burn (Upland‐Meadow)	−0.532	0.672	−2.141	0.514	1.000
Delta	LAOL	Burn (Upland‐Meadow)	0.040	0.586	−1.144	1.265	1.000
Delta	LECH	Burn (Upland‐Meadow)	0.008	0.608	−1.243	1.271	1.000
Delta	SYR3	Burn (Upland‐Meadow)	0.089	0.532	−0.956	1.223	1.000
Delta	TACH3	Burn (Upland‐Meadow)	−0.015	0.611	−1.281	1.238	1.000
Delta	ANUR	Burn (Upland‐Meadow)	−0.467	0.510	−1.593	0.413	1.000
Delta	APME	Burn (Upland‐Meadow)	0.180	0.413	−0.606	1.055	1.001
Delta	XECA	Burn (Upland‐Meadow)	−0.039	0.610	−1.328	1.186	1.000
Delta	LYGUS	Burn (Upland‐Meadow)	0.325	0.585	−0.707	1.641	1.000
Delta	ORSU	Burn (Upland‐Meadow)	0.080	0.544	−0.986	1.233	1.000
Delta	SYR4	Burn (Upland‐Meadow)	0.377	0.614	−0.664	1.810	1.000
Delta	Bomb4	Burn (Upland‐Meadow)	−0.253	0.559	−1.499	0.761	1.000
Delta	MEAN	Burn (Upland‐Meadow)	0.403	0.569	−0.555	1.692	1.000
Delta	LADI	Burn (Upland‐Meadow)	0.430	0.487	−0.413	1.504	1.000
Delta	MEMI	Burn (Upland‐Meadow)	−0.010	0.606	−1.273	1.236	1.000
Delta	HACO	Burn (Upland‐Meadow)	−0.417	0.568	−1.700	0.554	1.000
Delta	MORD1	Burn (Upland‐Meadow)	−0.596	0.678	−2.226	0.453	1.000
Delta	LASI2	Burn (Upland‐Meadow)	−0.295	0.624	−1.731	0.796	1.000
Delta	TACH1	Burn (Upland‐Meadow)	0.005	0.607	−1.253	1.257	1.000
Delta	CAED	Burn (Upland‐Meadow)	−0.110	0.587	−1.380	1.021	1.000
Delta	HALI	Burn (Upland‐Meadow)	−0.016	0.549	−1.135	1.113	1.000
Delta	HYEP	Burn (Upland‐Meadow)	−0.117	0.611	−1.445	1.073	1.000
Delta	CRAB1	Burn (Upland‐Meadow)	0.509	0.649	−0.528	2.053	1.000
Delta	OSMO	Burn (Upland‐Meadow)	−0.054	0.591	−1.297	1.129	1.000
Delta	Bomb6	Burn (Upland‐Meadow)	0.311	0.621	−0.763	1.746	1.000
Delta	PODAL	Burn (Upland‐Meadow)	0.486	0.642	−0.542	2.013	1.000
Delta	OSCO	Burn (Upland‐Meadow)	0.206	0.570	−0.851	1.479	1.000
Delta	GERON	Burn (Upland‐Meadow)	−0.307	0.649	−1.824	0.819	1.000
Delta	ANSA	Burn severity^2^, upland	−0.148	0.500	−1.282	0.763	1.000
Delta	CERAM	Burn severity^2^, upland	−0.162	0.517	−1.344	0.764	1.000
Delta	LAAN	Burn severity^2^, upland	0.200	0.498	−0.692	1.345	1.000
Delta	LAOL	Burn severity^2^, upland	−0.248	0.532	−1.506	0.647	1.000
Delta	LECH	Burn severity^2^, upland	−0.160	0.518	−1.348	0.769	1.000
Delta	SYR3	Burn severity^2^, upland	−0.039	0.453	−0.999	0.863	1.000
Delta	TACH3	Burn severity^2^, upland	−0.176	0.521	−1.383	0.749	1.001
Delta	ANUR	Burn severity^2^, upland	−0.218	0.453	−1.244	0.583	1.001
Delta	APME	Burn severity^2^, upland	0.099	0.368	−0.615	0.874	1.001
Delta	XECA	Burn severity^2^, upland	−0.164	0.513	−1.341	0.761	1.000
Delta	LYGUS	Burn severity^2^, upland	0.135	0.471	−0.752	1.169	1.000
Delta	ORSU	Burn severity^2^, upland	0.107	0.470	−0.799	1.135	1.000
Delta	SYR4	Burn severity^2^, upland	0.130	0.471	−0.754	1.174	1.000
Delta	Bomb4	Burn severity^2^, upland	0.189	0.492	−0.694	1.327	1.000
Delta	MEAN	Burn severity^2^, upland	0.148	0.457	−0.706	1.160	1.000
Delta	LADI	Burn severity^2^, upland	−0.065	0.392	−0.884	0.708	1.000
Delta	MEMI	Burn severity^2^, upland	−0.166	0.519	−1.359	0.761	1.000
Delta	HACO	Burn severity^2^, upland	−0.139	0.452	−1.146	0.695	1.000
Delta	MORD1	Burn severity^2^, upland	0.100	0.482	−0.832	1.151	1.000
Delta	LASI2	Burn severity^2^, upland	0.026	0.484	−0.959	1.038	1.000
Delta	TACH1	Burn severity^2^, upland	−0.167	0.514	−1.348	0.758	1.000
Delta	CAED	Burn severity^2^, upland	−0.152	0.501	−1.287	0.762	1.000
Delta	HALI	Burn severity^2^, upland	0.316	0.513	−0.535	1.539	1.001
Delta	HYEP	Burn severity^2^, upland	−0.189	0.516	−1.384	0.715	1.000
Delta	CRAB1	Burn severity^2^, upland	0.278	0.498	−0.572	1.452	1.000
Delta	OSMO	Burn severity^2^, upland	−0.223	0.520	−1.453	0.662	1.000
Delta	Bomb6	Burn severity^2^, upland	0.129	0.480	−0.774	1.200	1.000
Delta	PODAL	Burn severity^2^, upland	0.324	0.498	−0.510	1.492	1.000
Delta	OSCO	Burn severity^2^, upland	0.143	0.474	−0.741	1.197	1.000
Delta	GERON	Burn severity^2^, upland	0.058	0.498	−0.927	1.130	1.000
Sigma	Community	Intercept	1.176	0.307	0.606	1.833	1.002
Sigma	Community	DSS	0.720	0.184	0.404	1.123	1.000
Sigma	Community	Burn severity, meadow	0.244	0.069	0.142	0.407	1.000
Sigma	Community	Elevation (m)	0.270	0.076	0.154	0.446	1.000
Sigma	Community	Burn (Upland‐Meadow)	0.365	0.116	0.184	0.631	1.002
Sigma	Community	Burn severity^2^, upland	0.396	0.156	0.180	0.773	1.000
Sigma	Community	Habitat, upland	0.855	0.416	0.256	1.831	1.001
Sigma	Community	Floral abundance	0.627	0.279	0.224	1.280	1.001
Sigma	Community	Floral richness	0.490	0.226	0.194	1.051	1.001
Sigma	ANSA	Model	1.415	1.083	0.067	4.087	1.005
Sigma	CERAM	Model	0.808	0.695	0.033	2.598	1.003
Sigma	LAAN	Model	1.232	0.998	0.037	3.731	1.001
Sigma	LAOL	Model	0.983	0.844	0.030	3.114	1.003
Sigma	LECH	Model	0.962	0.823	0.029	3.031	1.002
Sigma	SYR3	Model	0.989	0.782	0.043	2.903	1.005
Sigma	TACH3	Model	0.958	0.822	0.036	3.061	1.002
Sigma	ANUR	Model	1.862	0.895	0.542	4.035	1.001
Sigma	APME	Model	1.585	0.627	0.677	3.124	1.003
Sigma	XECA	Model	1.066	0.847	0.048	3.152	1.002
Sigma	LYGUS	Model	2.034	1.156	0.264	4.807	1.003
Sigma	ORSU	Model	1.094	0.902	0.057	3.324	1.019
Sigma	SYR4	Model	1.063	0.880	0.035	3.307	1.006
Sigma	Bomb4	Model	1.455	1.044	0.083	3.960	1.002
Sigma	MEAN	Model	1.193	0.972	0.046	3.688	1.010
Sigma	LADI	Model	0.747	0.618	0.037	2.341	1.004
Sigma	MEMI	Model	1.015	0.843	0.042	3.147	1.001
Sigma	HACO	Model	1.510	1.013	0.055	3.865	1.007
Sigma	MORD1	Model	2.128	1.128	0.369	4.807	1.000
Sigma	LASI2	Model	1.081	0.888	0.044	3.307	1.009
Sigma	TACH1	Model	1.110	0.886	0.050	3.320	1.004
Sigma	CAED	Model	2.013	1.159	0.206	4.776	1.003
Sigma	HALI	Model	1.584	1.106	0.075	4.187	1.005
Sigma	HYEP	Model	2.589	1.396	0.329	5.914	1.003
Sigma	CRAB1	Model	1.409	1.116	0.064	4.152	1.007
Sigma	OSMO	Model	1.101	0.897	0.048	3.380	1.001
Sigma	Bomb6	Model	1.336	1.073	0.054	3.987	1.002
Sigma	PODAL	Model	1.126	0.899	0.046	3.343	1.000
Sigma	OSCO	Model	1.320	1.008	0.056	3.712	1.002
Sigma	GERON	Model	1.580	1.259	0.049	4.692	1.001
BP, species	ANSA	Model	0.559	0.497	0.000	1.000	1.000
BP, species	CERAM	Model	0.629	0.483	0.000	1.000	1.000
BP, species	LAAN	Model	0.434	0.496	0.000	1.000	1.000
BP, species	LAOL	Model	0.606	0.489	0.000	1.000	1.000
BP, species	LECH	Model	0.601	0.490	0.000	1.000	1.000
BP, species	SYR3	Model	0.350	0.477	0.000	1.000	1.000
BP, species	TACH3	Model	0.640	0.480	0.000	1.000	1.000
BP, species	ANUR	Model	0.389	0.487	0.000	1.000	1.000
BP, species	APME	Model	0.403	0.491	0.000	1.000	1.000
BP, species	XECA	Model	0.435	0.496	0.000	1.000	1.000
BP, species	LYGUS	Model	0.385	0.487	0.000	1.000	1.000
BP, species	ORSU	Model	0.391	0.488	0.000	1.000	1.000
BP, species	SYR4	Model	0.377	0.485	0.000	1.000	1.000
BP, species	Bomb4	Model	0.294	0.456	0.000	1.000	1.000
BP, species	MEAN	Model	0.315	0.464	0.000	1.000	1.000
BP, species	LADI	Model	0.347	0.476	0.000	1.000	1.000
BP, species	MEMI	Model	0.411	0.492	0.000	1.000	1.000
BP, species	HACO	Model	0.353	0.478	0.000	1.000	1.000
BP, species	MORD1	Model	0.443	0.497	0.000	1.000	1.000
BP, species	LASI2	Model	0.399	0.490	0.000	1.000	1.000
BP, species	TACH1	Model	0.456	0.498	0.000	1.000	1.000
BP, species	CAED	Model	0.504	0.500	0.000	1.000	1.000
BP, species	HALI	Model	0.297	0.457	0.000	1.000	1.000
BP, species	HYEP	Model	0.477	0.499	0.000	1.000	1.000
BP, species	CRAB1	Model	0.362	0.481	0.000	1.000	1.000
BP, species	OSMO	Model	0.578	0.494	0.000	1.000	1.000
BP, species	Bomb6	Model	0.362	0.480	0.000	1.000	1.000
BP, species	PODAL	Model	0.418	0.493	0.000	1.000	1.000
BP, species	OSCO	Model	0.345	0.476	0.000	1.000	1.000
BP, species	GERON	Model	0.374	0.484	0.000	1.000	1.000
BP, community	Community	Model	0.243	0.429	0.000	1.000	1.000
R	Community	Model	0.308	0.069	0.203	0.469	1.014

*Note*: Data were collected in 2016 and 2017 in the Sierra Nevada, California, following the 2014 King Fire. Beta shows the community‐level response to the covariate, and delta indicates the deviation of the species from the community response. The values reported in the text for species‐level response were derived from actual MCMC chains. See Table A3 for species code interpretation.

Abbreviations: BP, Bayesian *p*‐value; DSS, days since snowmelt; Sigma, standard deviation of random effect of site; *r*, negative binomial dispersion parameter.

**TABLE B4:** Posterior means, standard deviations (SD), and lower (2.50%) and upper (97.50%) limits of 95% Bayesian credible interval and convergence statistic (Rhat; <1.1 indicates convergence) of parameters from butterfly negative binomial generalized linear mixed models (separate model fit for each family).

Taxa	Parameter	Mean	SD	2.50%	97.50%	Rhat
Pieridae	Intercept	−0.086	0.525	−1.194	0.941	1.004
Pieridae	DSS	−1.007	0.212	−1.437	−0.602	1.000
Pieridae	Floral richness	−0.190	0.194	−0.566	0.198	1.001
Pieridae	Floral abundance	0.146	0.193	−0.230	0.529	1.001
Pieridae	Elevation (m)	−0.514	0.409	−1.383	0.263	1.001
Pieridae	Burn severity in meadow	0.776	0.456	−0.146	1.658	1.004
Pieridae	Habitat, upland	−2.710	0.839	−4.501	−1.148	1.004
Pieridae	Burn (Upland‐Meadow)	1.105	0.721	−0.231	2.608	1.003
Pieridae	Sigma	1.068	0.430	0.415	2.092	1.002
Pieridae	BP	0.406	0.491	0.000	1.000	1.000
Pieridae	*r*	0.586	0.149	0.349	0.930	1.000
Hesperiidae	Intercept	−1.981	0.685	−3.429	−0.717	1.009
Hesperiidae	DSS	−0.958	0.494	−2.062	−0.128	1.001
Hesperiidae	Floral richness	0.089	0.400	−0.702	0.893	1.001
Hesperiidae	Floral abundance	0.677	0.352	0.036	1.421	1.001
Hesperiidae	Elevation (m)	−1.122	0.603	−2.338	0.055	1.004
Hesperiidae	Burn severity in meadow	−0.464	0.719	−1.635	1.346	1.002
Hesperiidae	Habitat, upland	−3.261	1.296	−6.208	−1.163	1.006
Hesperiidae	Burn (Upland‐Meadow)	0.890	1.208	−1.930	3.033	1.000
Hesperiidae	Sigma	0.823	0.979	0.022	3.517	1.004
Hesperiidae	BP	0.356	0.479	0.000	1.000	1.001
Hesperiidae	*r*	1.288	8.094	0.111	1.931	1.128
Nymphalidae	Intercept	−0.434	0.535	−1.471	0.627	1.001
Nymphalidae	DSS	−0.283	0.175	−0.631	0.060	1.000
Nymphalidae	Floral richness	0.247	0.193	−0.120	0.636	1.001
Nymphalidae	Floral abundance	0.146	0.185	−0.207	0.522	1.000
Nymphalidae	Elevation (m)	−0.995	0.403	−1.850	−0.251	1.002
Nymphalidae	Burn severity in meadow	0.159	0.420	−0.686	0.982	1.003
Nymphalidae	Habitat (Upland‐Meadow)	−1.380	0.753	−2.914	0.037	1.001
Nymphalidae	Burn (Upland‐Meadow)	0.417	0.586	−0.765	1.562	1.002
Nymphalidae	Sigma	1.024	0.377	0.476	1.943	1.001
Nymphalidae	BP	0.378	0.485	0.000	1.000	1.000
Nymphalidae	*r*	0.535	0.153	0.303	0.898	1.001
Lycaenidae	Intercept	−1.438	0.626	−2.668	−0.221	1.001
Lycaenidae	DSS	0.076	0.406	−0.727	0.864	1.000
Lycaenidae	Floral richness	0.369	0.381	−0.356	1.148	1.000
Lycaenidae	Floral abundance	0.712	0.517	−0.210	1.832	1.001
Lycaenidae	Elevation (m)	−1.353	0.635	−2.735	−0.246	1.001
Lycaenidae	Burn severity in meadow	0.799	0.739	−0.709	2.249	1.001
Lycaenidae	Habitat (Upland‐Meadow)	−2.297	1.124	−4.750	−0.375	1.003
Lycaenidae	Burn (Upland‐Meadow)	1.323	1.128	−0.661	3.810	1.001
Lycaenidae	Sigma	0.677	0.645	0.026	2.379	1.002
Lycaenidae	BP	0.420	0.494	0.000	1.000	1.000
Lycaenidae	*r*	0.128	0.027	0.101	0.198	1.002

*Note*: Data were collected in 2016 in the Sierra Nevada, California, following the 2014 King Fire.

Abbreviations: BP, Bayesian *p*‐value; DSS, days since snowmelt; *r*, negative binomial dispersion parameter; Sigma, standard deviation of random effect of site.

**TABLE B5:** Posterior means, standard deviations (SD), and lower (2.50%) and upper (97.50%) limits of 95% Bayesian credible interval and convergence statistic (Rhat; <1.1 indicates convergence) of parameters from hummingbird negative binomial generalized linear mixed models.

	Mean	SD	2.50%	97.50%	Rhat
Intercept	−1.940	0.525	−3.069	−0.973	1.001
DSS	−0.843	0.234	−1.322	−0.403	1.001
Floral richness	0.375	0.160	0.071	0.704	1.003
Floral abundance	0.218	0.107	−0.001	0.425	1.001
Elevation (m)	−0.041	0.320	−0.660	0.614	1.001
Year	−0.735	0.412	−1.607	0.016	1.004
Burn severity in meadow	0.917	0.410	0.123	1.753	1.002
Habitat, upland	−1.899	0.971	−4.031	−0.188	1.010
Burn (Upland‐Meadow)	−0.093	0.605	−1.237	1.162	1.001
Burn severity^2^ in upland	−0.225	0.684	−1.540	1.148	1.002
Sigma	0.921	0.465	0.147	1.968	1.017
BP	0.304	0.460	0.000	1.000	1.002
*r*	19.824	27.661	0.459	92.567	1.025

*Note*: Data were collected in 2016 and 2017 in the Sierra Nevada, California, following the 2014 King Fire.

Abbreviations: BP, Bayesian *p*‐value; DSS, days since snowmelt; *r*, negative binomial dispersion parameter; Sigma, standard deviation of random effect of site.
